# ELKS1 Captures Rab6-Marked Vesicular Cargo in Presynaptic Nerve Terminals

**DOI:** 10.1016/j.celrep.2020.107712

**Published:** 2020-06-09

**Authors:** Hajnalka Nyitrai, Shan Shan H. Wang, Pascal S. Kaeser

**Affiliations:** 1 Department of Neurobiology, Harvard Medical School, Boston, MA 02115, USA; 2 Lead Contact

## Abstract

Neurons face unique transport challenges. They need to deliver cargo over long axonal distances and to many presynaptic nerve terminals. Rab GTPases are master regulators of vesicular traffic, but essential presynaptic Rabs have not been identified. Here, we find that Rab6, a Golgi-derived GTPase for constitutive secretion, associates with mobile axonal cargo and localizes to nerve terminals. ELKS1 is a stationary presynaptic protein with Golgin homology that binds to Rab6. Knockout and rescue experiments for ELKS1 and Rab6 establish that ELKS1 captures Rab6 cargo. The ELKS1-Rab6-capturing mechanism can be transferred to mitochondria by mistargeting ELKS1 or Rab6 to them. We conclude that nerve terminals have repurposed mechanisms from constitutive exocytosis for their highly regulated secretion. By employing Golgin-like mechanisms with anchored ELKS extending its coiled-coils to capture Rab6 cargo, they have spatially separated cargo capture from fusion. ELKS complexes connect to active zones and may mediate vesicle progression toward release sites.

## In Brief

Nyitrai et al. show that the small GTPase Rab6 is associated with mobile axonal cargo. ELKS1 is a broadly distributed presynaptic protein that captures cargo via binding to Rab6. Hence, nerve terminals have adapted a Golgin-like mechanism for vesicle capture and have spatially separated capture from exocytotic sites for regulation.

## Graphical Abstract

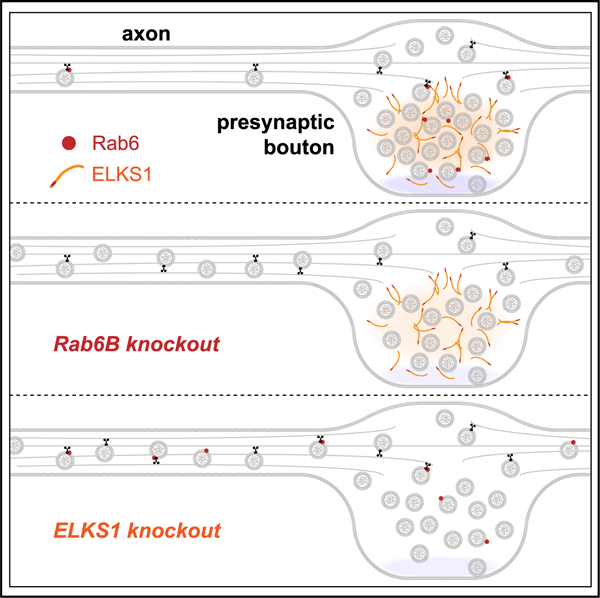

## INTRODUCTION

Neurons face great logistic challenges because they need to deliver secretory material to many presynaptic nerve terminals and over long axonal distances. Cell biological studies have revealed that different cellular compartments use tethering complexes at target sites to recognize and capture specific cargo (Cai et al., 2007; Munro, 2011). Rab GTPases are essential regulators of intracellular traffic. They are used as cargo-specific labels and act as molecular switches for cargo motility. In target compartments, they serve as recognition signals for tethering complexes, where cargo arrival is often linked to constitutive fusion (Hutagalung and Novick, 2011; Stenmark, 2009). In presynaptic nerve terminals, exocytosis is highly regulated (Jahn and Fasshauer, 2012; Südhof, 2013); therefore, cargo arrival must be separated from exocytosis. Despite the essential nature of delivering secretory material to nerve terminals, the cargo labels in axons and capturing mechanisms in nerve terminals are not well understood, and essential presynaptic Rabs have not been identified.

Of the more than 60 mammalian Rab genes, the most prominent presynaptic forms belong to the Rab3 family (Fischer von Mollard et al., 1990). Surprisingly, however, simultaneous knockout (KO) of all four Rab3 genes from mammalian neurons has no strong effect on synapse structure and function (Schlüter et al., 2004). Proteomic screens have identified a number of additional synapse-associated Rabs (Takamori et al., 2006; Wilhelm et al., 2014). Among these, Rab6 stands out because it is highly expressed in neurons (Opdam et al., 2000); is present on post-Golgi vesicles in non-neuronal cells, where it mediates capture followed by constitutive secretion (Fourriere et al., 2019; Grigoriev et al., 2007, 2011); and binds to the presynaptic protein family ELKS (Monier et al., 2002), which was named after the high content in glutamic acid (E), leucine (L), lysine (K), and serine (S) (Nakata et al., 1999). Rab6, expressed from two vertebrate genes (*Rab6A* and *Rab6B*), is one of only five Rabs that is evolutionarily conserved from yeast to humans (Pereira-Leal and Seabra, 2001). Neuronal Rab6 functions are not well understood, but at least overexpressed Rab6 is present in neurites in addition to its prominent Golgi localization (Schlager et al., 2014).

Rab6 and other Rabs bind to Golgins, large coiled-coil vesicle tethers located in the Golgi (Barr, 1999; Burguete et al., 2008; Fridmann-Sirkis et al., 2004; Hutagalung and Novick, 2011; Munro, 2011). While no Golgin is known to be present at synapses, there is striking homology between presynaptic ELKS proteins and Golgins (Munro, 2011), raising the hypothesis that ELKS may operate as a vesicle tether in nerve terminals similar to Golgin functions in Golgi trafficking.

ELKS proteins—also known as Rab6IP2 (Rab6-interacting protein 2), CAST (cytomatrix at the active zone associated structural protein), or ERC (abbreviation for ELKS, Rab6IP2, and CAST)—are large coiled-coil proteins thought to localize to vesicle fusion sites called active zones of presynaptic nerve terminals (Held and Kaeser, 2018; Monier et al., 2002; Ohtsuka et al., 2002; Wang et al., 2002). Ablation of the two mouse genes, *Erc1* and *Erc2*, or its fly homolog *brp*, leads to defects in neurotransmitter release (Dong et al., 2018; Held et al., 2016; Kittel et al., 2006; Liu et al., 2014) and is accompanied by impaired active zone structure, a role that is partially redundant with RIM (Rab3-interacting molecule) (Fouquet et al., 2009; Hagiwara et al., 2018; Held and Kaeser, 2018; Kittel et al., 2006; Wang et al., 2016; Wong et al., 2018), but its mechanisms in release have remained enigmatic. HeLa cells contain ELKS1 (also known as Rab6IP2 or CAST2; see Held and Kaeser [2018] for an overview of nomenclature) as part of an adhesion complex for microtubules at the cell cortex. There, Rab6-containing vesicles undergo constitutive fusion upon arrival (Grigoriev et al., 2007, 2011; Lansbergen et al., 2006), but the molecular mechanisms through which ELKS and Rab6 contribute to constitutive fusion have remained incompletely understood.

Together, these findings raise the hypothesis that ELKS operates in the capture of arriving Rab6-tagged cargo in presynaptic nerve terminals. But this model is inconsistent with ELKS localization at the active zone because microtubules are typically not active zone or membrane anchored, but they often pass the nerve terminal in the back (Gordon-Weeks et al., 1982; Schrod et al., 2018). It is also not compatible with the mechanisms of Rab6 in constitutive secretion because these roles rely on plasma membrane anchoring of microtubules at fusion sites (Grigoriev et al., 2007, 2011; Lansbergen et al., 2006). Lastly, it is unclear whether Rab6 is present in axons. Alternative models are that neuronal Rab6 is important for the biogenesis of vesicular cargo in the soma or that it has no role in the generation or targeting of axonal cargo.

Here, we establish that ELKS1 proteins form an extended network throughout the nerve terminal and capture Rab6-positive axonal cargo in presynapses of cultured hippocampal neurons. This capturing mechanism can be translocated to mitochondria by artificially localizing ELKS or Rab6 to mitochondria. Our work leads to a model in which neurons have repurposed a mechanism from constitutive secretion for their highly regulated exocytosis. ELKS1-mediated capture allows for spatially separating cargo capture inside the nerve terminal from cargo exocytosis at the active zone of the target membrane. Presynaptic protein assemblies that span the nerve terminal and contain ELKS may connect to the active zone and enable vesicle delivery to release sites and presynaptic regulation.

## RESULTS

### Rab6 Is Highly Expressed in Neurons and Is Present in Nerve Terminals

**Figure 1.**
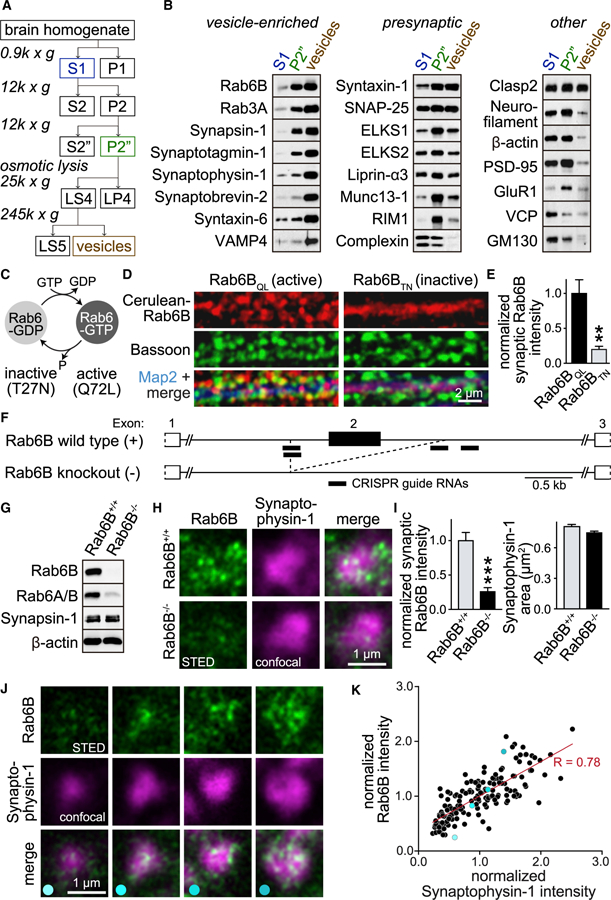
Rab6 Partially Localizes to Presynaptic Nerve Terminals (A) Schematic of the vesicle fractionation. (B) Representative western blots detecting various proteins in S1, P2′′, and vesicle fractions. (C) Schematic of the Rab6 cycle and point mutations that mimic active or inactive states. (D and E) Representative confocal images (D) and quantification (E) of Rab6 levels in synapses of hippocampal neurons transduced with lentiviral Cerulean-Rab6B and immunostained for Cerulean-Rab6B (with anti-GFP antibodies), Bassoon (to mark synapses), and Map2 (to mark dendrites). Fluorescent intensities within Bassoon ROIs were normalized to the average Rab6B_QL_ intensity. Rab6B_QL_, n = 8 images/3 independent cultures; Rab6B_TN_, n = 7/3 (each image containing 640 Bassoon objects on average). (F) CRISPR KO strategy for constitutive Rab6B ablation. (G) Example western blots of brain homogenates from 90-day-old Rab6B^+/+^ or Rab6B^−/−^ littermate mice. (H and I) Example images (H) and quantification (I) of immunostained synapses of cultured hippocampal neurons from Rab6B^+/+^ or Rab6B^−/−^ mice. Rab6B (acquired by STED microscopy) levels within Synaptophysin-1 (acquired by confocal microscopy) ROIs were quantified and normalized to Rab6B^+/+^. Rab6B^+/+^, n = 19 images/3 independent cultures; Rab6B^−/−^, n = 21/3 (each image containing 30 Synaptophysin-1 objects on average). (J and K) Example images (J) and quantification (K) of Synaptophysin-1 and Rab6B levels in Rab6B^+/+^ hippocampal neurons. The correlation between signal intensities was assessed using Pearson’s correlation analyses, n = 155 synapses/3 independent cultures. Summary data are means ± SEM, **p < 0.01, ***p < 0.001, analyzed by Student’s t test. For synaptosome fractionations and analyses of Rab6B_QL_ and Rab6B_TN_ levels and Rab6^−/−^ mutant mice, see Figure S1.

Mammals have two paralogous *Rab6* genes, the ubiquitously expressed *Rab6A* and the brain-specific *Rab6B*, and primates have an additional retrogene, *Rab6C* (Opdam et al., 2000; Pereira-Leal and Seabra, 2001; Young et al., 2010). We hypothesized that Rab6 may in part be present in nerve terminals because it is expressed in brain (Opdam et al., 2000), binds to presynaptic ELKS (Monier et al., 2002), and has been identified in presynaptic proteomes (Takamori et al., 2006; Wilhelm et al., 2014). We focused on Rab6B because it is the prominent Rab6 in brain (Opdam et al., 2000). Rab6 was enriched in mouse brain relative to other tissues, as assessed by western blotting (Figure S1A), and its expression increased from postnatal days P1 to P90. Cortical brain lysates were fractionated into synaptosomes (Figures S1B and S1C) or vesicle fractions in which synaptic vesicles dominate (Figures 1A and 1B). Rab6B was highly enriched in synaptosomes (Figure S1C) and in the vesicle fraction (Figure 1B). GM130, a Golgin that is localized to the Golgi apparatus, failed to enrich in these fractions (Figures 1B and S1C).

Rab6, like other Rab GTPases, cycles between active (GTP-bound) and inactive (GDP-bound) states (Figure 1C), which determine its association with vesicular compartments (Stenmark, 2009) and can be mimicked with point mutations (Opdam et al., 2000; Stenmark, 2009). To test whether Rab6 localizes state dependently to synapses, we cultured hippocampal neurons from wild-type mice and expressed Cerulean-Rab6B_QL_ (Q72L, active state) or Cerulean-Rab6B_TN_ (T27N, inactive state) using lentiviruses. We fixed neuronal cultures at day *in vitro* (DIV) 14, stained them with anti-GFP antibodies (which recognize Cerulean-Rab6), co-stained for Bassoon to mark synapses, and acquired images using confocal microscopy. Rab6B_QL_ was punctate and partially overlapped with Bassoon (Figures 1D, 1E, and S1D). In contrast, Rab6B_TN_ was diffusely localized throughout neurites but not enriched in synapses.

We next assessed the localization of endogenous Rab6B using constitutive Rab6B KO (Rab6B^−/−^) mice as negative controls. Rab6B^−/−^ mice were generated by CRISPR-mediated deletion of exon 2 of the *Rab6B* gene (Figure 1F). The offspring ratio in litters of Rab6B^+/−^ parents showed a normal Mendelian distribution (Figure S1E), and the Rab6B protein (Figure 1G) and its mRNA (Figure S1F) were removed in Rab6B^−/−^ mice. Rab6B^−/−^ also removed most signal for an antibody that recognizes Rab6A and Rab6B (Figures 1G, S1G, and S1H), confirming that Rab6B is the dominant Rab6 isoform in brain (Opdam et al., 2000).

To visualize the distribution of endogenous Rab6B, we used super-resolution microscopy in cultured Rab6B wild-type (Rab6B^+/+^) and Rab6B^−/−^ neurons. Rab6B (imaged by stimulated emission depletion (STED) microscopy) formed small puncta that overlapped with the synaptic vesicle marker Synaptophysin-1 (imaged by confocal microscopy). This synaptic Rab6B signal was strongly reduced in Rab6B^−/−^ neurons (Figures 1H and 1I), as was the prominent Golgi signal (Figure S1H), establishing signal specificity. Finally, in Rab6B^+/+^ neurons, the synaptic Rab6B intensities positively correlated with levels of Synaptophysin-1 (Figures 1J and 1K), supporting that the Rab6B signal arises from its synaptic localization. Together, these data establish that Rab6B is present in axons, partially localizes to nerve terminals, and associates with vesicles in addition to its known Golgi localization.

### Rab6B KO Leads to Enhanced Axonal Cargo Deposition away from Synapses

**Figure 2.**
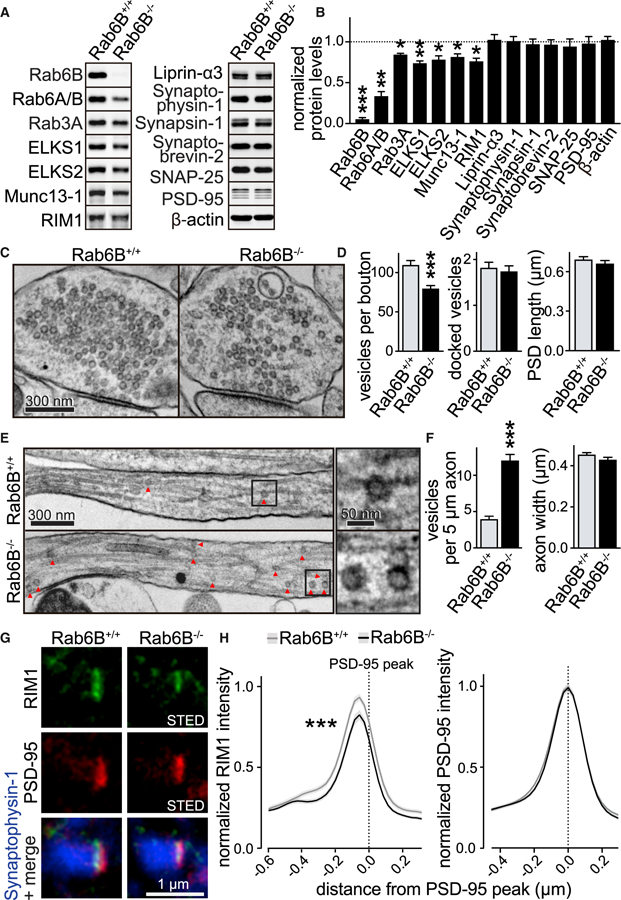
Rab6B KO Impairs Presynaptic Structure and Leads to Increased Axonal Cargo Accumulation (A and B) Example western blots (A) and quantification of protein levels (B) with fluorescent secondary antibodies of hippocampal neurons. Levels were normalized to Rab6B^+/+^ in each culture, Rab6B^+/+^, n = 3 independent cultures; Rab6B^−/−^, n = 3. (C and D) Example electron microscopic images (C) and quantification (D) of synapses of high-pressure frozen hippocampal neurons. Rab6B^+/+^, n = 144 synapses/2 independent cultures; Rab6B^−/−^, n = 158/2. (E and F) Example images of axons (E) and analysis of axonal structure and axonal vesicles (F) of the experiment shown in (C) and (D). Rab6B^+/+^, n = 207 axonal segments/2 independent cultures; Rab6B^−/−^, n = 167/2. (G and H) Example STED images (G) and quantification (H) of side-view synapses of hippocampal neurons. RIM1 and PSD-95 signals were acquired by STED microscopy, and Synaptophysin-1 by confocal microscopy. (H) Normalized RIM1 intensity profiles (shaded rectangle), quantified as described in Figure S2D, with the 0-μm position set to the PSD-95 peak, and negative values are distances from the PSD-95 peak toward the presynapse. Rab6B^+/+^, n = 76 synapses/3 independent cultures; Rab6B^−/−^, n = 77/3. Summary data are means ± SEM, *p < 0.05, **p < 0.01, ***p < 0.001, analyzed by Student’s t test (B, D, and F) or two-way ANOVA (for RIM1 in H: genotype ***, distance ***, interaction n.s.; for PSD-95 in H: n.s. for all). For additional STED and electron microscopic analyses, see Figure S2.

We next assessed Rab6B^−/−^ neurons by quantitative fluorescent western blotting. Ablation of Rab6B was accompanied by some-whatdecreased total levels of active zone proteins, while other presynaptic or postsynaptic proteins were unaffected (Figures 2A and 2B). We hypothesized that Rab6B is involved in the delivery of material to nerve terminals. Previous studies reported that axonal delivery defects can result in aberrant accumulation of cargo in axons away from synapses (Barber et al., 2017; Miller et al., 2005). We employed either high-pressure freezing followed by freeze substitution (Figures 2C–2F and S2A) or chemical fixation (Figures S2B and S2C) of hippocampal neurons and assessed synaptic and axonal ultrastructure using transmission electron microscopy. The number of vesicles per bouton was decreased by ~25% in Rab6B^−/−^ KO nerve terminals, and there was a striking ~3-fold increase in the number of vesicles in axons outside of presynaptic boutons. We call these vesicles ‘‘axonal vesicles,’’ as opposed to synaptic vesicles that are present in boutons. The number of docked synaptic vesicles, the size of the postsynaptic density, and the axon width were unaffected. We also found that nerve terminal size was somewhat decreased, while there was an increase in the area covered by endosomal structures in boutons (Figure S2A). In conclusion, axonal vesicles are much more frequently present in axons of Rab6B^−/−^ neurons, and there are changes in presynaptic ultrastructure that are consistent with impaired cargo delivery.

Since there was a decrease in total active zone protein levels (Figures 2A and 2B), we asked whether Rab6 removal impairs their delivery and active zone incorporation. We used two-color STED microscopy (Figure S2D), as we described before (de Jong et al., 2018; Wong et al., 2018). In brief, we labeled synapses with antibodies against synaptic vesicles (imaged by confocal microscopy), the postsynaptic density marker PSD-95 (imaged by STED microscopy), and the active zone protein RIM1 (imaged by STED microscopy). We selected ‘‘side-view’’ synapses, in which PSD-95 formed a bar aligned to one edge of the vesicle cloud, and positioned a rectangular region of interest (ROI) perpendicular to the axis of the PSD-95 bar across the synapse. Within this region, we assessed peak localization and signal intensity of PSD-95 and RIM1. RIM1 peak intensities were reduced in Rab6B^−/−^ synapses, but the remaining signal was localized at the correct position (Figures 2G and 2H). Using a similar approach, we found that Bassoon peak intensities remained unchanged (Figures S2E and S2F). Together, these findings suggest that ablation of Rab6B impairs the delivery or active zone incorporation of at least some presynaptic material. This could occur through impaired genesis, transport, or capture of presynaptic cargo or through impaired active zone anchoring because of structural active zone defects.

### ELKS1 and ELKS2 Bind to Rab6 with a Sequence Motif near the C Terminus

If presynaptic ELKS captures Rab6-tagged cargo, the two proteins should bind to one another, as indicated by earlier studies (Monier et al., 2002). We addressed ELKS1 and ELKS2 (see Held and Kaeser [2018] and Figure S3A for an overview of ELKS protein variants) binding to Rab6, including mapping of the exact binding sites, and generated ELKS mutants that fail to bind to Rab6. First, we performed affinity purifications from brain lysates using glutathione S-transferase (GST)-tagged Rab proteins as baits. We found that active Rab6A and Rab6B (Rab6A_QL_ and Rab6B_QL_) interacted with endogenous ELKS1 and ELKS2, but not with any other of the tested presynaptic proteins (Figure S3B), and this interaction was not mediated by the known Rab3-interacting protein RIM1 (Figure S3C). To determine ELKS isoform specificity, we transfected HEK293T cells with ELKS variants and assessed Rab6 binding. We tested all key isoforms (Figure S3A) that are expressed from separate genes (ELKS1 versus ELKS2) as N-terminal promoter variants (ELKSα versus ELKSβ) or as C-terminal splice variants (ELKSA versus ELKSB). We found that each ELKS variant bound to active Rab6, but not to inactive Rab6 or to Rab3 (with the exception of a weak binding observed between active

**Figure 3.**
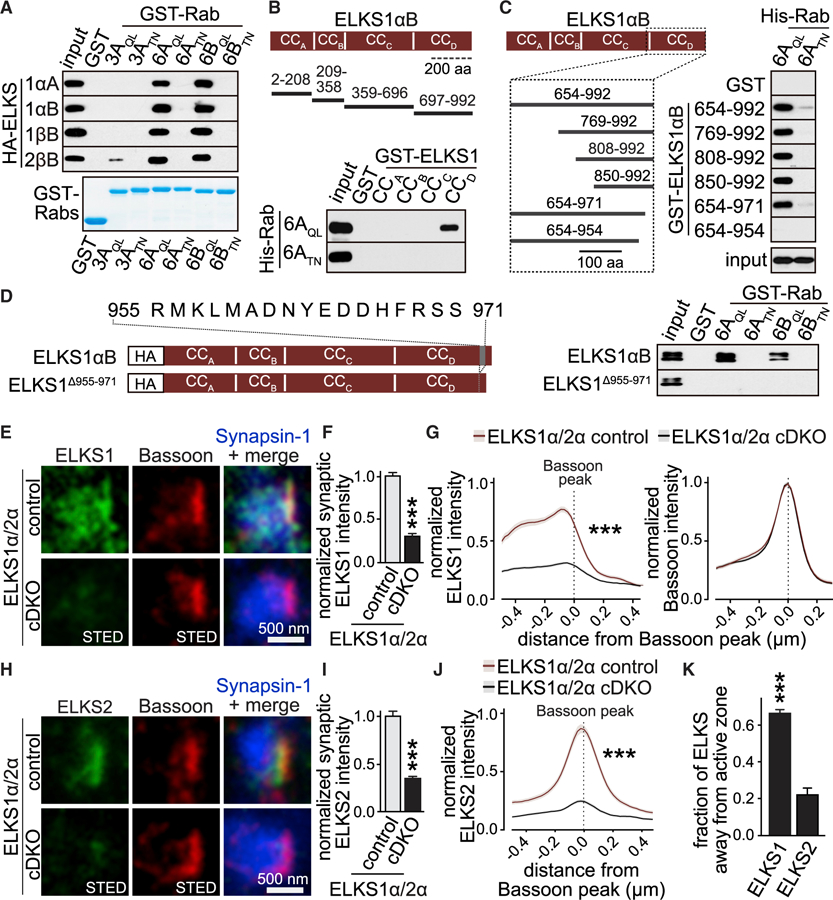
Defining the Rab6 Binding Site and the Widespread Presynaptic Localization of ELKS1 (A–D) Example western blots of GST-affinity purifications using various ELKS and Rab6 proteins. (A) GST-Rabs were used to pull down ELKS from lysates of transfected HEK293T cells, bound ELKS was detected by hemagglutinin (HA) antibodies, and purified GST-fusion proteins are shown on a Coomassie stained gel below. GST-ELKS coiled-coil regions covering ELKS1 (B) or shorter fragments of ELKS1 CC_D_ (C) were used to pull down purified His-Rab6A. (D) GST-Rab6 was used to pull down ELKS1αB or ELKS1^∆955-971^ from lysates of transfected HEK293T cells, and bound ELKS was detected by HA antibodies. Input lanes contain 5% of total input; each experiment was repeated three independent times except for ELKS1αA, ELKS1αB, and ELKS2αA conditions in (A) (performed once), (B) (two independent repeats), and the Rab6A condition in (D) (performed once). (E–G) Example images (E) and quantification (F and G) of the subsynaptic localization of ELKS1 in side-view hippocampal synapses, assessed by STED microscopy as described in Figure S2D, but with Bassoon as an active zone marker. Mice with floxed alleles for ELKS1α and ELKS2α (Liu et al., 2014) were cultured and infected with a lentivirus expressing Cre recombinase (to generate ELKS1α/2α cDKO neurons) or a recombination-deficient Cre virus (to generate ELKS1α/2α control neurons). (F) ELKS1α/2α control, n = 15 images/3 independent cultures; ELKS1α/2α cDKO, n = 15/3, (each image containing 60 Bassoon objects on average); (G) ELKS1α/2α control, n = 73 synapses/3 independent cultures; ELKS1α/2α cDKO, n = 71/3. (H–J) As in (E)–(G), but for ELKS2, Bassoon localization was assessed as in (G) but is not shown. (I) ELKS1α/2α control, n = 15/3; ELKS1α/2α cDKO, n = 14/3 (each image containing 45 Bassoon objects on average). (J) ELKS1α/2α control, n = 66/3; ELKS1α/2α cDKO, n = 63/3. (K) Quantification of the fraction of the synaptic ELKS1 or ELKS2 that does not colocalize with the active zone marker Bassoon in STED images of ELKS1α/2α control synapses. ELKS1 and ELKS2: n = 15 images/3 independent cultures (each image containing 52 Bassoon objects on average). Rab3A and ELKS2βB; Figure 3A). Hence, the interaction site lies within a sequence that is shared between these ELKS variants. Summary data are means ± SEM, ***p < 0.001, analyzed by Student’s t test (F, I, and K) or two-way ANOVA ([G] ELKS1: genotype ***, distance ***, interaction n.s., Bassoon: n.s. for all; [J] genotype ***, distance ***, interaction ***, Holm-Sidak’s post-test on peak intensity: ***). For ELKS protein isoforms, additional affinity binding assays, and immunostainings, see Figure S3.

To establish direct binding between the two proteins and to map the Rab6-binding sites on ELKS, we used recombinant affinity purification assays with GST-coupled fragments of ELKS1αB. We split ELKS1αB into its four coiled-coil domains, CC_A_–CC_D_ (Held et al., 2016), and assessed binding to purified His-Rab6. ELKS1-CC_D_ bound to active Rab6A, but not to the inactive mutant (Figure 3B). We further narrowed the binding site by creating shorter fragments and found that a 17-amino-acid region (amino acids 955–971) of ELKS1-CC_D_ was required for Rab6A binding (Figure 3C). Rab6A and Rab6B bound to the same site on ELKS, the same region mediated binding of Rab6 to ELKS1 and ELKS2, and binding was also selective for wild-type Rab6 with non-hydrolysable GTP (Figures S3D–S3G). To test for necessity of these 17 amino acids in full-length ELKS, we transfected either ELKS1αB or ELKS1 lacking this motif (ELKS1^∆955-971^) into HEK293T cells and performed affinity purifications from the cell lysates. ELKS1^∆955-971^ failed to bind to Rab6A or Rab6B (Figure 3D), while ELKS1αB efficiently bound to the active mutants of each Rab6 isoform. In summary, active, GTP-bound Rab6A and Rab6B bind to ELKS1 and ELKS2, and a 17-amino-acid sequence motif near the C terminus is necessary for the ELKS-Rab6 interaction. This area of the protein is highly conserved in vertebrates and similar in *C. elegans* (Figure S3E), but absent in the fly homolog Bruchpilot that lacks ELKS homology in its C-terminal half (Held and Kaeser, 2018; Monier et al., 2002).

### ELKS1 but Not ELKS2 Is Positioned to Capture Rab6 Cargo

ELKS proteins have been described as active zone proteins due to their biochemical interactions with other active zone proteins and their localization assessed with antibodies against ELKS2 or Bruchpilot (Fouquet et al., 2009; Kittel et al., 2006; Ohtsuka et al., 2002; Wang et al., 2002; Wong et al., 2018). However, ELKS1 active zone localization has not been experimentally established. ELKS could not mediate cargo capture if it were strictly localized at active zones because most presynaptic material is delivered via microtubular transport, and microtubules are typically not in close proximity to the active zone (Gordon-Weeks et al., 1982; Schrod et al., 2018). We used STED microscopy to assess the localization of ELKS1 and ELKS2 and used established conditional double KO (cDKO) of ELKS1α and ELKS2α (ELKS1α/2α cDKO) as negative controls (Figures S3H and S3I) (Held et al., 2016; Liu et al., 2014). In these STED experiments (Figures 3E–3K), we used Bassoon as an active zone marker within a synapse, while in previous confocal microscopic experiments with lower resolution, Bassoon is used as a marker for synapses (Figures 1D and 1E). In side-view STED images of synapses, the subsynaptic localization of the two ELKS proteins was strikingly different (Figures 3E–3K and S3J). ELKS2 was confined to the active zone, and its peak precisely colocalized with Bassoon (Figures 3H–3J). In contrast, ELKS1 was widely distributed throughout nerve terminals with no clear peak, and its mild average peak was shifted 100 nm toward the inside of the nerve terminal relative to Bassoon (Figures 3E–3G and S3J). We next analyzed ELKS signals independent of side-view synapse selection. We generated synaptic ROIs defined by Synapsin-1 and active zone ROIs defined by Bassoon and quantified the fraction of synaptic ELKS1 or ELKS2 that was not at the active zone (Figure 3K). While ELKS2 strongly overlapped with Bassoon, the majority of ELKS1 was away from the active zone. In summary, while both ELKS1 and ELKS2 bind to Rab6 and are primarily presynaptic, only ELKS2 is restricted to the active zone. In contrast, ELKS1 proteins are widespread in nerve terminals and positioned such that they could capture arriving cargo.

### ELKS1α/β KO Leads to Enhanced Axonal Cargo Deposition away from Synapses

**Figure 4.**
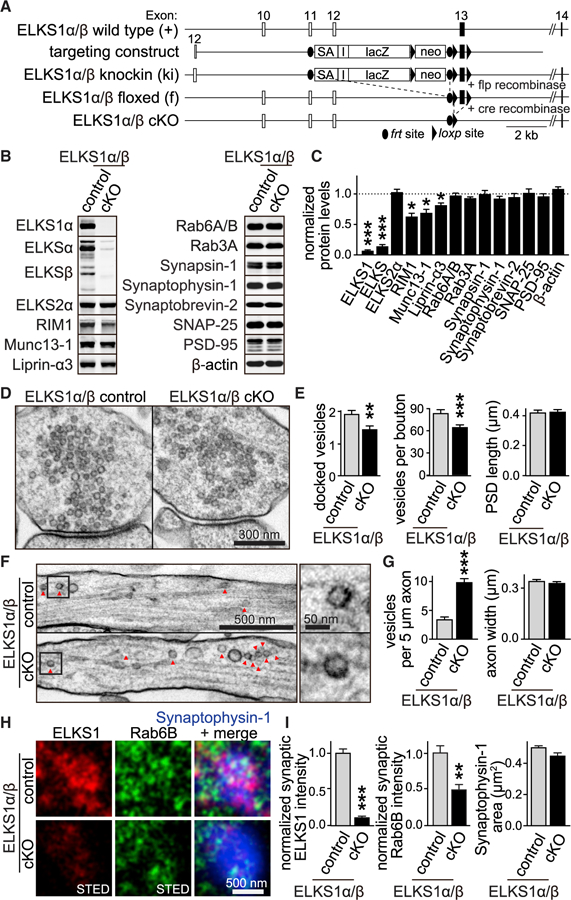
ELKS1α/β KO Impairs Presynaptic Structure and Leads to Increased Axonal Cargo Accumulation (A) *Erc1* gene targeting strategy to conditionally ablate ELKS1α/β. (B and C) Example western blots (B) and quantification of protein levels (C) with fluorescent secondary antibodies of hippocampal neurons of mice with floxed alleles for ELKS1α/β, infected with a lentivirus expressing Cre recombinase (to generate ELKS1α/β cKO neurons) or a recombination-deficient Cre virus (to generate ELKS1α/β control neurons). Levels were normalized to the levels in ELKS1α/β control in each culture; n = 3 independent cultures. (D and E) Example electron microscopic images (D) and quantification (E) of synapses of high-pressure frozen neurons. ELKS1α/β control, n = 134 synapses/2 independent cultures; ELKS1α/β cKO, n = 148/2. (F and G) Example images of axons (F) and analysis of axonal structure and axonal vesicles (G) of the experiment shown in (D) and (E). ELKS1α/β control, n = 209 axonal segments/2 independent cultures; ELKS1α/β cKO, n = 221/2. (H and I) Example STED images (H) and quantification (I) of synaptic ELKS1 and Rab6. Quantifications in (I) show normalized intensities within Synaptophysin-1 ROIs, ELKS1α/β control, n = 25 images/3 independent cultures; ELKS1α/β cKO, n = 20/3 (each image containing 30 Synaptophysin-1 objects on average). Summary data are means ± SEM, *p < 0.05, **p < 0.01, ***p < 0.001, analyzed by Student’s t test. For additional analyses of ELKS1α/β cKO mice and electron microscopic experiments, see Figure S4.

ELKS1α/2α cDKO neurons have upregulated expression of ELKS1β (Liu et al., 2014), which binds to Rab6 (Figure 3A). To address potential ELKS1 cargo capture functions without this limitation, we analyzed ELKS1 conditional KO (cKO) mice with simultaneous ablation of ELKS1α and ELKS1β (Figures 4A and S4A–S4D). The cKO ELKS1α/β mice were generated by homologous recombination in embryonic stem cells. The original mutant allele (ki) had a splice-acceptor cassette 5′ of exon 13 that disrupted ELKS1α and ELKS1β expression (Figure 4A). This mutation was lethal in homozygotes (Figure S4A), similar to constitutive ELKS1α KOs (Liu et al., 2014). The conditional ELKS1α/β floxed (f) mice, generated by flp recombinase-mediated deletion of the splice acceptor cassette, expressed ELKS1, and offspring of heterozygote crossings survived at normal Mendelian ratios (Figure S4B).

We cultured homozygous floxed ELKS1α/β hippocampal neurons and infected them with lentiviruses expressing Cre recombinase (to generate ELKS1α/β cKO neurons) or a truncated, inactive version of Cre (to generate ELKS1α/β control neurons). Quantitative fluorescent western blotting confirmed that ELKS1α and ELKS1β were removed, and ELKS1α/β removal led to a modest but significant decrease of active zone protein levels (Figures 4B and 4C). This is strikingly similar to Rab6B^−/−^ neurons (Figure 2B). Key similarities were also present in synaptic and axonal ultrastructure (Figures 4D–4G and S4E–S4G): the number of vesicles per bouton was reduced, a robust accumulation of axonal vesicles was observed, and endosome areas were also increased. These effects were similar in high-pressure frozen (Figures 4F and 4G) or glutaraldehyde fixed cultures (Figures S4F and S4G), when cultures were infected with Cre virus at DIV1 (Figure S4G) or DIV5 (Figures 4F, 4G, and S4F), or when ELKS1α and ELKS2α were removed instead of ELKS1α/β (Figure S4H). In addition to this phenocopy of Rab6B^−/−^ neurons, there was a modest reduction in vesicle docking, which could either be secondary to the loss of vesicle capture or due to functions of ELKS1 downstream of vesicle capture.

We next tested whether ELKS1 ablation leads to reduced synaptic Rab6, as predicted for a capturing mechanism. Compellingly, synaptic Rab6B antibody labeling was decreased by more than 50% (Figures 4H, 4I, and S4I), while overall neuronal Rab6 levels were unchanged (Figures 4B and 4C), supporting the working model in which Rab6-tagged cargo is captured in nerve terminals by ELKS1. The observation that total levels of Rab6 and other vesicular proteins are unchanged suggests a defect in cargo capture rather than cargo generation.

### Binding of Rab6 to ELKS1 Is Necessary for Cargo Delivery

**Figure 5.**
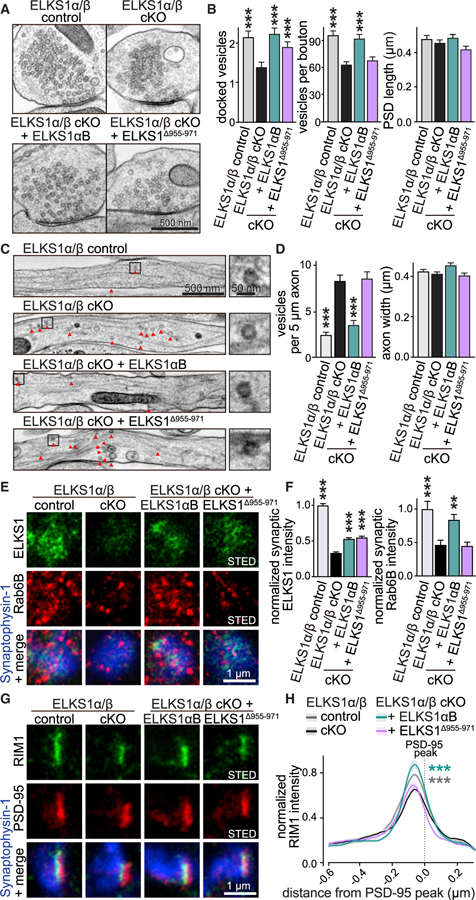
Rab6 Binding of ELKS1 Is Necessary to Reverse ELKS1α/β KO Phenotypes (A and B) Example electron microscopic images (A) and quantification (B) of synapses of high-pressure frozen ELKS1α/β control and ELKS1α/β cKO neurons, and ELKS1α/β cKO neurons rescued with ELKS1αB or ELKS1^∆955-971^ using lentiviral expression. ELKS1α/β control, n = 82 synapses/2 independent cultures; ELKS1α/β cKO, n = 116/2; ELKS1α/β cKO + ELKS1αB, n = 103/2; ELKS1α/β cKO + ELKS1^∆955-971^, n = 105/2. (C and D) Example images of axons (C) and analysis of axonal structure and axonal vesicles (D) of theexperiment shown in (A) and (B). ELKS1α/β control, n = 143 axonal segments/2 independent cultures; ELKS1α/β cKO, n = 165/2; ELKS1α/β cKO + ELKS1αB, n = 133/2; ELKS1α/β cKO + ELKS1^∆955-971^, n = 148/2. (E and F) Example STED images (E) and quantification (F) of synaptic ELKS1 and Rab6 signals. Synpatophysin-1 staining (acquired by confocal microscopy) was used to define ROIs. ELKS1α/β control, n = 78 synapses/3 independent cultures; ELKS1α/β cKO, n = 73/3; ELKS1α/β cKO + ELKS1αB, n = 75/3; ELKS1α/β cKO + ELKS1^∆955-971^, n = 76/3. (G and H) Example STED images (G) and quantification (H) of the RIM1 signals in side-view synapses. ELKS1α/β control, n = 63/3; ELKS1α/β cKO, n = 61/3; ELKS1α/β cKO + ELKS1αB, n = 53/3; ELKS1α/β cKO + ELKS1^∆955-971^, n = 60/3. Summary data are means ± SEM, **p < 0.01, ***p < 0.001, analyzed by one-way ANOVA (B, D, and F, all ***) followed by Holm-Sidak’s post-tests (p values against ELKS1α/β cKO reported in B, D, and F) or by two-way ANOVA (H, genotype ***, distance ***, interaction n.s.) followed by Holm-Sidak’s post-tests (p values against ELKS1α/β cKO reported in H). For analysis of overall rescue expression levels and PSD-95 peak position and levels, see Figure S5.

If ELKS1 captures Rab6-tagged cargo, binding of Rab6 to ELKS1 should be important for capture. To test this, we expressed ELKS1αB or ELKS1^∆955-971^ (that is unable to bind Rab6; Figure 3D) in ELKS1α/β cKO neurons using lentiviruses for rescue (Figure 5). Expression levels of both rescue constructs were similar but lower than the expression of endogenous ELKS1 (Figure S5A). The neurons were subjected to either high-pressure freezing followed by electron microscopy or to immunostaining followed by STED microscopy.

ELKS1αB re-expression restored vesicle docking and the number of vesicles in boutons, and it reversed the accumulation of axonal vesicles assessed by electron microscopy (Figures 5A–5D and S5B). ELKS1^∆955-971^ was unable to rescue the vesicle numbers in boutons or the accumulation of axonal vesicles, but it restored vesicle docking, at least partially.

We next analyzed rescue using STED microscopy (Figures 5E–5H and S5C). We generated Synaptophysin-1 ROIs and assessed ELKS1 and Rab6B levels within them. ELKS1αB and ELKS1^∆955-971^ localized similarly to synapses, but their synaptic levels were lower than those of endogenous ELKS1, consistent with the results obtained from western blotting (Figures 5E, 5F, and S5A). ELKS1 but not ELKS1^∆955-971^ rescued synaptic Rab6B levels. When we analyzed RIM1 in side-view synapses, we found that the peak of RIM1 was mildly but significantly reduced upon ELKS1 ablation (Figures 5G and 5H), similar to Rab6B KO (Figures 2G and 2H). ELKS1αB rescue restored this deficit to levels above control, while ELKS1^∆955-971^ failed to do so. In all conditions, PSD-95 peak levels and localization remained unaffected (Figure S5C). These experiments support the model that ELKS1 captures Rab6 by a direct interaction and indicate that effects of Rab6 deletions are caused by loss of this interaction at synapses.

### ELKS1 Is Stably Localized to Synapses while Axonal Rab6 Is Mobile

**Figure 6.**
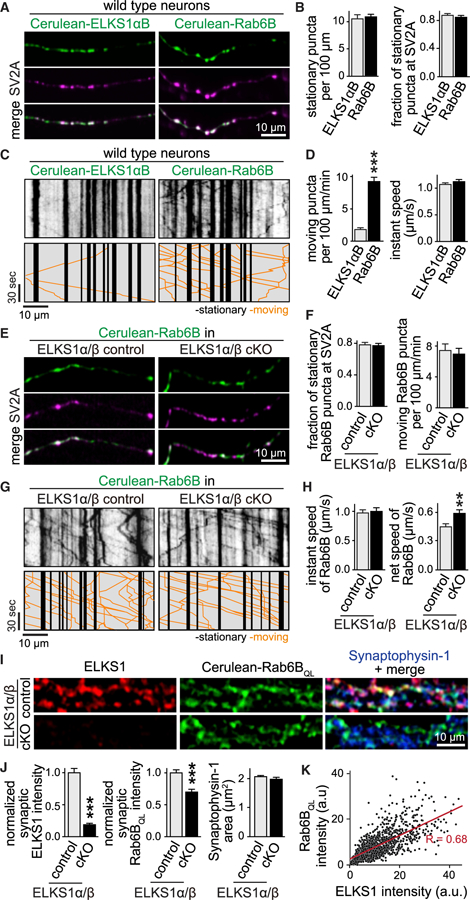
Presynaptic ELKS1 Captures Mobile Rab6B (A–D) Example images of axons (A ), example kymographs of a different set of axons (C), and quantifications (B and D) of live wide-field imaging of wild-type neurons transfected with Cerulean-ELKS1αB or Cerulean-Rab6B. The number of stationary puncta (A and B) or moving puncta in kymographs (C and D) was quantified in time-lapse images. tdTomato-SV2A was co-transfected to identify synapses. Stationary puncta (B) and flux of moving puncta (D, left) were first averaged per cell, and the average across cells is shown: ELKS1αB, n = 34 cells/3 independent cultures; Rab6B, n = 34/3. Speed of moving puncta (D, right) is an average of individual moving events: ELKS1αB, n = 105 moving puncta/3 independent cultures; Rab6B, n = 340/3. (E–H) Example images of axons (E), example kymographs of a different set of axons (G), and quantifications (F and H) of live imaging in ELKS1α/β control and ELKS1α/β cKO neurons transfected with Cerulean-Rab6B and tdTomato-SV2A. The number of stationary Cerulean-Rab6B puncta colocalizing with SV2 (F, left), the number of moving Cerulean-Rab6B puncta (F, right), and the instant and net speed of the moving puncta in kymographs (H) was quantified. (F) ELKS1α/β control, n = 18 cells/3 independent cultures; ELKS1α/β cKO, n = 27/3; (H) control, n = 291 moving puncta/3 independent cultures; cKO, n = 302/3. (I and J) Example confocal images (I) and quantification (J) of synaptic levels of Cerulean-tagged, transduced Rab6B_QL_ (detected with anti-GFP antibodies) in hippocampal neurons. ELKS1α/β control, n = 24 images/3 independent cultures; ELKS1α/β cKO, n = 22/3 (each image containing 35 Synaptophysin-1 objects on average). (K) Pearson’s correlation analysis of Cerulean-Rab6B_QL_ and ELKS1 fluorescent intensities of the ELKS1α/β control condition shown in (E) and (F). n = 870 synapses/3 independent cultures. Summary data are means ± SEM, **p < 0.01, ***p < 0.001, analyzed by Student’s t test. For correlation of endogenous Rab6B and ELKS1 and Rab6A capture at synapses, see Figure S6.

If ELKS1 captures Rab6B-tagged cargo, synapses with more ELKS1 should contain more Rab6. We examined Rab6 and ELKS1 signal intensities in Synaptophysin-1 ROIs and found a strong positive correlation (Figures S6A and S6B). The model of ELKS1-Rab6-based cargo capture further implies that ELKS1 is a stationary presynaptic protein, while Rab6 is mobile. An alternative possibility would be that ELKS1 and Rab6 are co-trafficked on the same cargo. To distinguish between these possibilities, we sparsely expressed Cerulean-ELKS1αB or Cerulean-Rab6B in wild-type neurons and co-transfected tdTomato-SV2A to mark synapses. We then performed live imaging of axonal ELKS1 and Rab6B using wide-field fluorescence microscopy and generated kymographs to assess their dynamic behavior (Figures 6A–6D). Both ELKS1αB and Rab6B had ~1 stationary punctum every 10 μm axon, and ~80% of those puncta colocalized with SV2A (Figure 6B), indicating that they were synaptic. The two proteins, however, had very different dynamic behaviors. Moving ELKS1αB puncta were rare, with only ~1 moving object per 100 μm of axon per minute. In contrast, Rab6B was highly mobile, with ~8 moving objects per 100 μm of axon per minute (Figure 6D). Hence, while ELKS1 is stationary at synapses, Rab6B is highly dynamic and frequently transported in axons in addition to its localization to synapses.

Finally, the ELKS1-Rab6-capturing model predicts that the synaptic recruitment of Rab6, but not its axonal transport, is impaired upon ablation of ELKS1. Rab6B exhibited normal instant speeds in ELKS1α/β cKO neurons, suggesting normal axonal traffic (Figures 6G and 6H). Furthermore, the net speed of individual trafficking events, consisting of movement and pause time, was increased upon ELKS1α/β KO, suggesting that Rab6 pauses less frequently in the absence of ELKS. We then tested presynaptic capture of Cerulean-Rab6B_QL_ and Cerulean-Rab6A_QL_ in ELKS1α/β cKO neurons, as these proteins are efficiently targeted to synapses (Figures 1C–1E). Presynaptic accumulation of Rab6_QL_ was decreased upon ablation of ELKS1α/β, and in control synapses Rab6_QL_, intensities were positively correlated with endogenous ELKS1 intensities (Figures 6I–6K and S6C–S6E). Hence, Rab6 capture is impaired upon ablation of ELKS1. We conclude that ELKS1 is stationary and Rab6 is mobile, and Rab6 stabilization at synapses is dependent on the presynaptic presence of ELKS1.

### Hijacking of Rab6-ELKS1 Interactions to Mistarget Vesicles or Mitochondria

**Figure 7.**
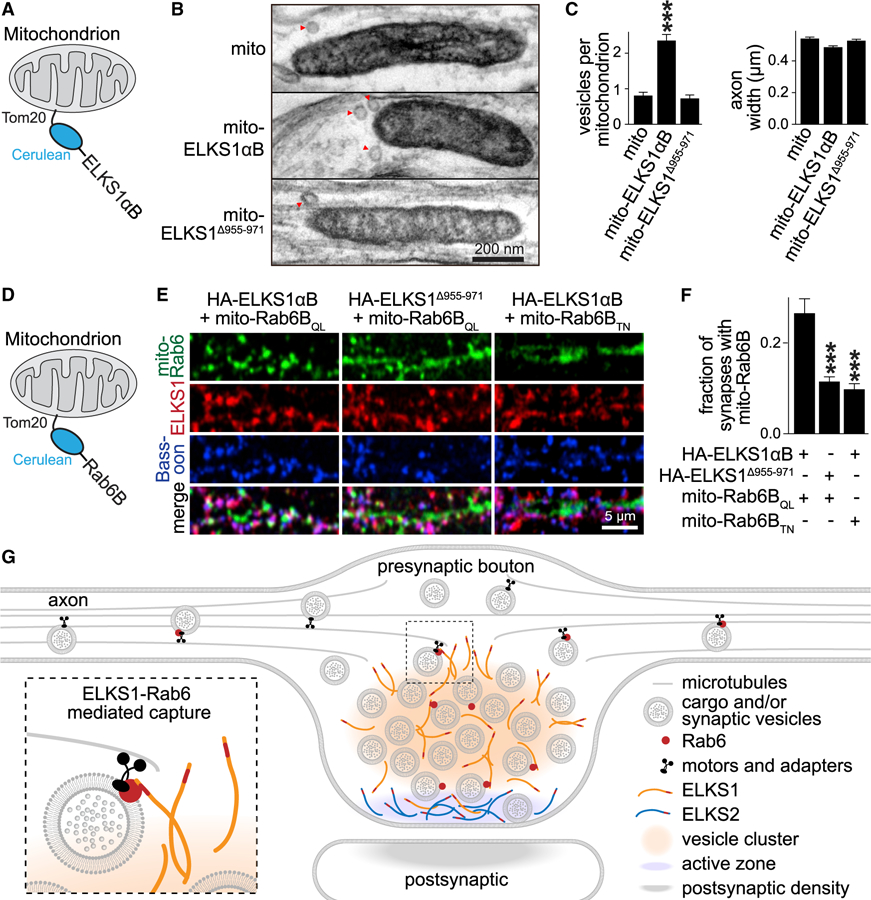
Hijacking the Rab6-ELKS1-Capturing Mechanism and Working Model (A–C) Schematic of the experiment (A), representative electron microscopic images (B), and quantification of vesicles within 70 nm (see Method Details for a justification of this criterion) of the mitochondrial membrane (C) upon artificial targeting of ELKS1 to mitochondria in wild-type hippocampal neurons were transduced with lentiviruses. Mito (control), n = 198 mitochondria/2 independent cultures; mito-ELKS1αB, n = 208/2; mito-ELKS1^∆955-971^, n = 197/2. (D–F) Schematic of the experiment (D), representative confocal images (E), and quantification (F) of the fraction of synapses with mito-Rab6-tagged mitochondria. Neurons were transduced with two lentiviruses to express mito-Rab6B and HA-tagged ELKS1 and immunostained for mito-Rab6 (GFP antibodies), HA-ELKS1 (HA antibodies), and Bassoon (to label synapses). HA-ELKS1αB + mito-Rab6B_QL_, n = 19 images/3 independent cultures; HA-ELKS1^∆955-971^ + mito-Rab6B_QL_, n = 21/3; HA-ELKS1αB + mito-Rab6B_TN_, n = 18/3 (each image containing 60 Bassoon objects on average). (G) Working model of the mechanism by which synapse-anchored ELKS1 captures axonal Rab6 cargo. Rab6 is associated with trafficking cargo, and ELKS1 is localized to nerve terminals. A direct interaction between Rab6 and a C-terminal sequence element (highlighted in red) in ELKS1 mediates cargo capture (inset, bottom left). Summary data are means ± SEM, ***p < 0.001, analyzed by one-way ANOVA (C, condition ***; F, condition ***), followed by Holm-Sidak’s post-test (p values indicated to mito-tag alone in C or to ELKS1αB + mito-Cerulean-Rab6B_QL_ in F). For assessment of mitochondrial localization of mito-tagged proteins and their expression levels, see Figure S7.

If ELKS1 is sufficient for Rab6 cargo capture, one might be able to transfer this mechanism to other cellular structures. To test this, we mistargeted ELKS1βB or Rab6B to mitochondria by fusing their N termini to the transmembrane region of the outer leaflet mitochondrial protein Tom20 (Kanaji et al., 2000), followed by Cerulean for visualization, referring to these proteins as mito-ELKS1αB (Figure 7A) or mito-Rab6B (Figure 7D).

Mito-ELKS1αB was efficiently expressed in neurons by lentiviral transduction and localized to mitochondria (Figures S7A and S7B). To assess whether ELKS1 is sufficient to capture Rab6 cargo, we tested whether mito-ELKS1αB or Rab6-binding-deficient mito-ELKS1^∆955-971^ leads to accumulation of vesicular cargo on the mitochondrial surface by electron microscopy (Figures 7A–7C). Remarkably, mito-ELKS1αB expression led to a ~3-fold increase in the number of vesicles (diameter ≤ 50 nm) associated with the mitochondrial surface, compared to expression of the mito-tag alone, and this increase was absent for mito-ELKS1^∆955-971^ (Figures 7B and 7C).

We next expressed active mito-Rab6B_QL_ or inactive mito-Rab6B_TN_ in neurons using lentiviruses and co-expressed either HA-ELKS1αB or HA-ELKS1^∆955-971^ from an independent lentivirus. The proteins were efficiently expressed in neurons, and mito-Rab6 localized to mitochondria (Figures S7C and S7D). Neurons were fixed at DIV15 and immunostained with anti-GFP antibodies to visualize the Cerulean-tagged mito-Rab6B, anti-HA antibodies for HA-ELKS1, and anti-Bassoon antibodies to mark synapses. We then used confocal microscopy to quantify the fraction of synapses that contained Rab6B-tagged mitochondria. Co-expression of mito-Rab6B_QL_ and HA-ELKS1αB led to a 2-fold increase in the fraction of synapses with mitochondria, compared to inactive Rab6B or ELKS1 that does not bind to Rab6 (Figures 7D–7F). Hence, the Rab6-ELKS1 interaction is capable of mediating cargo capture in these experiments, even if the cargo or the target compartment are mitochondria, simply by mistargeting Rab6 or ELKS1 onto them, respectively.

## DISCUSSION

### A Model for Presynaptic Cargo Capture

How presynaptic material is transported along axons and captured in nerve terminals has remained poorly understood. In most intracellular traffic, Rab GTPases operate as master organizers to control cargo mobility during transport and cargo tethering at target sites. Presynaptic nerve terminals are perhaps the most tightly controlled and highly regulated secretory compartments, but essential Rabs have not been identified thus far. Here, we establish a model in which a subset of presynaptic cargo is labeled by Rab6 and captured at synapses by ELKS1 (Figure 7G), and we rely on four lines of evidence. First, Rab6 is associated with mobile axonal cargo and with presynapses, while ELKS1 is stably localized in nerve terminals and broadly distributed within them. Second, KO of Rab6B or ELKS1 leads to accumulation of axonal cargo and to loss of some presynaptic material. KO of ELKS1, in addition, leads to reduced presynaptic Rab6 levels. Third, ELKS1 KO phenotypes are restored with rescue, but rescue is ineffective when the 17 amino acids necessary for ELKS1-Rab6 binding are removed. Finally, the capturing mechanism can be transferred to mitochondria by mistargeting ELKS1 or Rab6 to them. It is unexpected that Rab6 mediates cargo delivery to synapses. Previous work has established that Rab6 controls trafficking between Golgi compartments (Echard et al., 1998; Feldmann et al., 1995; Monier et al., 2002) or mediates constitutive secretion of arriving cargo at exocytotic sites of cortical microtubules (Grigoriev et al., 2007, 2011; Lansbergen et al., 2006). Our data establish that neurons have re-purposed this mechanism for their highly regulated exocytotic machine by spatially separating cargo arrival from cargo exocytosis. We propose that an ELKS protein network forms a bridge between the two processes that allows for cargo delivery to secretory sites and for regulation.

### ELKS as a Cargo Tether at the Target Site

We propose that the functions and mechanisms of ELKS are similar to those of Golgins in the Golgi apparatus. Golgins form a loosely organized array of tentacles that is anchored to the Golgi membrane (Munro, 2011). They contain coiled-coil stretches that form extended, homodimeric antenna-like tethers and contain Rab-binding sites to capture vesicles. ELKS proteins indeed share key properties of Golgins in addition to the sequence homology: their coiled-coils form elongated homo- and heterodimers (Deguchi-Tawarada et al., 2004; Sala et al., 2019), and they bind to Rab6 (Figure 3; Monier et al., 2002). The key difference between ELKS and Golgins is their localization. While Golgins are anchored to Golgi membranes, ELKS proteins are predominantly presynaptic in neurons (Liu et al., 2014). Within nerve terminals, we find a striking difference in the distribution of ELKS1 and ELKS2. ELKS2 is tightly clustered at the active zone, but ELKS1 is widespread in nerve terminals. Because microtubules are distant from the active zone within a nerve terminal, this widespread localization of ELKS1 enables cargo capture.

Golgins are membrane anchored, which allows for cargo tethering at the Golgi surface. How could ELKS1 be anchored? ELKS proteins bind to several active zone proteins, including RIM, Liprin-α, and Bassoon, and they homodimerize with one another and form heterooligomers (Deguchi-Tawarada et al., 2004; Held and Kaeser, 2018; Ko et al., 2003; Ohtsuka et al., 2002; Sala et al., 2019; Takao-Rikitsu et al., 2004; Wang et al., 2002). Through interactions with active zone proteins and oligomerization, it is possible that ELKS1 forms a filamentous network throughout the nerve terminal that is anchored at the active zone. Such an ELKS protein mesh could capture arriving cargo and may contribute to vesicle movement toward the active zone through progressive handing down of a captured cargo. The N-terminal and central coiled-coil sequences of ELKS bind to the active zone proteins Bassoon and Liprin-α, interactions that could potentially anchor the ELKS protein network. The same area of ELKS binds to LL5β (Grigoriev et al., 2007, 2011; Lansbergen et al., 2006). LL5β has not been identified in nerve terminals, but it binds to phospholipid membranes and is localized to exocytotic sites for constitutive secretion in a complex that anchors microtubule plus-ends (Grigoriev et al., 2007, 2011; Lansbergen et al., 2006). Hence, data from constitutive secretion generally support the model that the N-terminal half of ELKS mediates its anchoring.

An alternative model is that ELKS participates in liquid-phase condensates that tether vesicles. ELKS1 was recently shown to undergo phase transitions and to form liquid droplets within cells, and the N-terminal half alone is sufficient to form phase condensates (Sala et al., 2019). In this model, ELKS is dynamically tethered by its property to from a liquid phase or by participating in a Synapsin-based liquid phase that tethers synaptic vesicles (Milovanovic et al., 2018). Its C-terminal coils may form tentacles that reach out of the phase to capture Rab6 cargo. Interactions with ELKS2 or other active zone proteins, some of which undergo phase separation as well (Wu et al., 2019), may further account for the dynamic nature of vesicle clustering and vesicle progression toward release sites. Interestingly, even Golgins form liquid-phase condensates (Rebane et al., 2020), and liquid-liquid-phase separation may be an organizational principle for vesicle tethering in the Golgi apparatus. Ultimately, an overarching and speculative model arises in which phase separation is a general mechanism through which vesicle tethering and traffic is organized throughout cells.

### Rab6 for the Targeting of Presynaptic Cargo

Rab GTPases are conserved molecular switches for cargo mobility and exocytosis from yeast to mammals. Surprisingly, however, in KOs of the most abundant presynaptic Rabs, Rab3A-3D, no defects in synapse structure and only mild impairments in neurotransmission were detected (Schlüter et al., 2004). Hence, it has remained uncertain whether other Rabs fulfill these roles and whether Rab proteins are essential for synapses. Here, we present the unexpected finding that Rab6 is present on mobile axonal cargo and mediates cargo capture at synapses. At sites for constitutive exocytosis in HeLa cells, cortical microtubules are anchored via plus-end tethering complexes, and Rab6 links cargo transport and arrival to constitutive exocytosis (Fourriere et al., 2019; Grigoriev et al., 2007, 2011; Lansbergen et al., 2006). How could Rab6 contribute synaptic secretion where release is highly regulated? We propose that the ELKS1 protein network serves to spatially separate cargo arrival from exocytosis, re-purposing this Rab6-dependent mechanism.

Given that synapses are complex secretory machines with multiple synaptic vesicle pools and other cargo, it is unlikely that a single mechanism accounts for all capture. Indeed, while we observe a robust increase in axonal cargo deposition away from synapses, loss of cargo in nerve terminals is partial upon Rab6B KO. Hence, other mechanisms must exist. What could such redundant mechanisms be? A trivial explanation is that Rab6A partially compensates. This appears unlikely because Rab6B is the dominant brain isoform and Rab6A was not upregulated upon Rab6B KO, and future gene ablation studies should assess this. One alternative mechanism could operate through regulation of anterograde transport by Arl-8, a small Arf-like GTPase. In *C. elegans*, Arl-8 promotes anterograde axonal transport and inhibits cargo deposition, and this activity is antagonized by JNK (c-Jun N-terminal kinase) (Klassen et al., 2010; Wu et al., 2013). A second alternative mechanism couples cargo unloading to increased microtubule dynamics in nerve terminals compared to other axonal areas (Guedes-Dias et al., 2019). GTP-tubulin is enriched by the increased dynamics, and binding of the motor KIF1A to GTP-tubulin is weaker than to GDP-tubulin, and this weakened interaction enhances cargo unloading in nerve terminals. Ultimately, it appears likely that at least three mechanisms cooperate for cargo delivery to nerve terminals: (1) cargo capture via molecular interactions between Rab GTPases and presynaptic proteins (this study); (2) local suppression of anterograde cargo transport within nerve terminals (Klassen et al., 2010; Wu et al., 2013); and (3) weakened local cargo-microtubule interactions (Guedes-Dias et al., 2019). Loss of any one mechanism leads to partial impairments in the delivery of presynaptic material because the remaining mechanisms, and perhaps additional unknown mechanisms, continue to support cargo delivery.

A related question concerns the composition of Rab6 cargo. This is currently unclear, and Rab6 cargo could be involved in the transport of active zone material, consistent with the loss of some active zone proteins upon Rab6B KO, or other synaptic material. This raises a broader unanswered question, namely, how homogeneous the content of axonal cargo packages is and whether there are definable subclasses of axonal cargo. Models of co-transport of synaptic vesicle and active zone precursors, separate transport of active zone precursors, and variable origins of presynaptic precursors have been proposed (Ahmari et al., 2000; Emperador-Melero and Kaeser, 2020; Emperador-Melero et al., 2018; Shapira et al., 2003; Vukoja et al., 2018). Our electron microscopic data indicate that Rab6 labels small clear vesicular cargo, and STED microscopy and western blotting suggest that active zone protein capture is partially mediated by ELKS1 and Rab6. Hence, Rab6 cargo may contain at least some active zone proteins, and these proteins may be transported on a synaptic vesicle-like cargo. This cargo may be distinct, identical, or overlapping with synaptic vesicle precursors, and Rab6 may label other cargo as well. Ultimately, the knowledge that Rab6 labels an axonal cargo provides a molecular handle for future studies to assess the molecular composition and properties of this cargo.

## STAR★METHODS

### RESOURCE AVAILABILITY

#### Lead Contact

Further information and requests for resources and reagents should be directed to and will be fulfilled by the Lead Contact, Pascal S. Kaeser (kaeser@hms.harvard.edu).

#### Materials Availability

Plasmids generated for this study will be shared without restrictions. Antibodies generated for this study are exhaustible and will be shared as long as they are available. The newly described mouse lines for knockout of Rab6B or ELKS1 are available through MMRRC, and will also be shared upon request within the limits of the respective material transfer agreements.

#### Data and Code Availability

This study did not generate datasets or code.

### EXPERIMENTAL MODEL AND SUBJECT DETAILS

All animal experiments were performed according to protocols approved by the Harvard University Animal Care and Use Committee. Constitutive Rab6B knockout mice (C57BL/6N-*Rab6b*^*em1(IMPC)J*^/Mmucd, RRID:MMRRC_049340-UCD, also called CRISPR_JR28993) were obtained as homozygous adults from the Jackson Laboratory (Stock# 028993). The line was generated at the Jackson Laboratory as part of the NIH KOMP initiative by CRISPR/Cas9 gene editing, targeting the *Rab6B* gene in zygotes, which resulted in the deletion of exon 2 spanning an area of ~200 bp. The following primers were used for genotyping: GAGCCAGCCTT TAAGTGCGCGT and CCTGCCTCTTCAAAAGATCC that produce a 466-bp band for a wild-type allele and a 288 bp band for knockout allele. The line was maintained by mating heterozygote Rab6B^+/−^ mice, and only Rab6B^+/+^ and Rab6B^−/−^ littermates were used to generate neuronal cultures or brain lysates for experiments. The conditional ELKS1α/β mice (C57BL/6N-*A*^*tm1Brd*^ *Erc1*^*tm1a(EUCOMM)Hmgu*^/BayMmucd; RRID:MMRRC_041523-UCD) were acquired as frozen sperm from the Mutant Mouse Resource and Research Center (MMRRC) at University of California at Davis, an NIH-funded strain repository. The mice were produced at Baylor College of Medicine as part of the BaSH Consortium for the NIH Common Fund program (Skarnes et al., 2011). The mice were generated with a targeting vector using a knockout-first strategy, with a reporter-tag inserted upstream of exon 13 of the *Erc1* gene, and the reporter cassette contained an frt site followed by a splice acceptor, a lacZ sequence, and a loxP site. This first loxP site was followed by a neomycin resistance cassette, a second frt site and a second loxP site. A third loxP site was inserted downstream of exon 13. Homologous recombination was performed in embryonic stem cells (knockin allele in Figure 4A), followed by injection of ES cell clone HEPD0819_1_A01 into C57BL/6J-*Tyr*^*c-Brd*^ blastocysts. This allele never produced surviving homozygous ELKS1α/β^ki/ki^ mice and is likely a loss-of-function allele, consistent with previous publications that showed that constitutive ELKS1α knockout is lethal (Liu et al., 2014). The ELKS1α/β ki mice were genotyped using the primer pair CCGTTGATTCTGAACAGTGTAGG (forward) and CCGAACATTGGAAGTAGGTAATCC (reverse), which produced a 375-bp band in wild-type and no band for ki, and the primer pair GGGATCTCATGCTGGAGTTCTTCG (forward) and the reverse primer as above, which produced no band in wild-type and a 745-bp band for ki.

To generate the conditional ELKS1α/β floxed line, the frt-flanked neomycin cassette was removed by crossing the ELKS1α/β^ki/ki^ mice to mice that express *Flp* recombinase under a β-actin promoter (Dymecki, 1996). The ELKS1α/β floxed line was bred to homozygosity and the *Flp* transgene was outbred. The floxed line was genotyped with two reactions: primer pair GCCCAAACAGAAGTT GACCGTC (forward) and CTTTGGACTCTCTAGAACATAGC (reverse) produced a 360-bp band in wild-type and no band in floxed allele; the primer pair using the same forward oligo with GAACTGATGGCGAGCTCAGACC (reverse) produces a 390-bp band in floxed and no band in wild-type allele. ELKS1α/2α conditional double knockout mice with floxed alleles for *Erc1* (RRID:IMSR_ JAX:015830) and *Erc2* (RRID:IMSR_JAX:015831) to remove ELKS1α and ELKS2α, but not β-ELKS proteins produced by either gene, were previously described (Kaeser et al., 2009; Liu et al., 2014). RIM1α/β constitutive knockout mice were previously described (Kaeser et al., 2008) and were generated by germline recombination of a ‘‘floxed’’ conditional knockout allele available at the Jackson laboratories (STOCK *Rims1*^*tm3Sud*^/J, JAX:015832).

### METHOD DETAILS

#### Neuronal cell culture and lentivirus production

Primary mouse hippocampal cultures were prepared from newborn pups (P0-P1) of either sex as described (Held et al., 2016; Liu et al., 2014) on sapphire (for high-pressure freezing experiments) or glass (all other experiments) coverslips in 24-well plates. For all experiments, neuronal cultures were harvested at DIV14–16. Lentiviruses were produced in HEK293T cells and were used immediately after harvest. HEK293T cells were grown in DMEM with 10% (v/v) bovine serum and 1% (v/v) penicillin/streptomycin and were passaged every 1–3 days up to 20 passages for maintenance. To generate lentiviruses, HEK293T cells were transfected using a standard Ca^2+^-phosphate transfection protocol with 3^rd^ generation lentiviral packaging plasmids (pVSVG [pHN120108], pRRE [pHN120109], pREV [pHN120110]) and a lentiviral plasmid (pFSW containing the human Synapsin promoter and a cDNA specific to the experiment). After 24 h, the culture medium was exchanged to neuronal cell growth medium and virus production was allowed to proceed for another 24 h. The culture medium from HEK293T cells was harvested 48 h after transfection, centrifuged for 5 min at 700 x g to pellet cell debris. EGFP-Cre-expressing viruses (produced by co-transfection of the lentiviral packaging plasmids with plasmid pHN131014) and inactive mutants of cre (pHN131015) were added to the cultured neurons at DIV5 unless otherwise noted (130 μL per well). Infection rates were monitored by nuclear EGFP expression, and only cultures where no uninfected neurons were detected were used for analysis. Rescue viruses (HA-ELKS1αB, pHN161031, or HA-ELKS1^∆955-971^, pHN170936) were added at DIV3 (200 μL per well). For Rab6 expression experiments (Figures 1D, 6I–6K, and S6C–S6E), cultures were infected at DIV5 with 100 μL of lentiviral solution expressing Cerulean-tagged Rab6B_QL_ (pHN160705), Rab6B_TN_ (pHN160706), or Rab6A_QL_ (pHN160326). For mitochondrial mistargeting of ELKS1 (Figure 7B), wild-type neuronal cultures were infected at DIV3 with 200 μL lentiviral solutions expressing either mito-ELKS1αB (pHN161033) or mito-ELKS1αB^∆955-971^ (pHN190429), or were infected at DIV5 with 50 μL lentiviral solution expressing the mito-tag alone (pHN161037, also called Tom20-Cerulean, infection volume was adjusted because the small tag expressed more efficiently). For mitochondrial mistargeting of Rab6B (Figure 7E), wild-type neuronal cultures were infected at DIV3 with 200 μL lentiviral solutions expressing either HA-ELKS1αB (pHN161031) or HA-ELKS1^∆955-971^ (pHN170936), followed by a second independent infection at DIV5 with 130 μL lentiviral solution expressing either mito-Rab6B_QL_ (pHN181203) or mito-Rab6B_TN_ (pHN181204). For control experiments to determine the effectiveness of the mito-tag (Figure S7), wild-type neuronal cultures were infected at DIV5 with 50 μL of mitoDsRed (pHN161038) to mark endogenous mitochondria and were simultaneously infected with 130 μL of one of the following lentiviruses: Cerulean-tagged ELKS1αB (pMYW12018), mito-ELKS1αB (pHN161033), mito-Rab6B_QL_ (pHN181203), or mito-Rab6B_TN_ (pHN181204).

#### Antibody generation

A new pan-ELKS antiserum (HM1083) was raised in rabbits against a GST-fusion protein of the ELKS1αB fourth coiled-coil domain (CC_D_, plasmid pLB12025) expressed and purified from bacteria by standard procedures described under protein expression. The immunogen was similar to a previously generated pan-ELKS antibody (P224 in Wang et al., 2002). The GST-fusion protein was purified and eluted from the beads with 10 mM glutathione for 3 h at 4°C. After overnight dialysis in PBS at 4°C, the protein solution was snap-frozen in ethanol/dry ice and submitted to Cocalico Biologicals for immunization in rabbits. Rabbits were given booster injections every two weeks, and bleeds were collected every three weeks. Crude sera were screened using western blot against protein samples harvested from cultured neurons. Sera with the highest immunoreactivity (bleeds 3 to 6) were used at 1:2,000 dilution. Isoform specificity of HM1083 (Figure S4C) was tested in lysates from HEK293T cells transfected with plasmids expressing the various isoforms (pLB12010, pLB12011, pLB12013, and pLB14065). Notably, HM1083 appears to have stronger reactivity with ELKS1 than ELKS2 (Figures 4 and S4), apparently different from P224 (Kaeser et al., 2009; Liu et al., 2014; Wang et al., 2002).

#### Cortical synaptosome and vesicle fractionations

Mouse cortices from 6-week old mice were homogenized in 10% w/v homogenizing buffer (320 mM sucrose, 4 mM HEPES pH 7.4, and 1x Sigma Protease Inhibitor Cocktail for mammalian cells) with 3 × 10 strokes on ice with a glass-Teflon homogenizer and centrifuged at 1,000 x g for 10 min at 4°C. The pellet (P1) was separated from the supernatant (S1), and S1 was centrifuged at 12,500 x g for 15 min at 4°C. The supernatant (S2) was collected and stored, and the pellet (P2) was used for further fractionation. Synaptosome preparation was carried out as described before (Liu et al., 2018). The P2 pellet was resuspended in 1 mL homogenizing buffer, and was layered on top of a sucrose gradient (5 mL 1.2 M sucrose on the bottom and 5 mL 0.8M sucrose in a round-bottom ultracentrifuge tube), and was centrifuged at 141,000 x g for 1.5 h at 4°C using a swing-bucket rotor (SW41). The synaptosome layer (1.5 ml) was collected from the interface of the two sucrose layers and analyzed with western blotting. The cortical homogenate, S1, S2, and P2 fractions were diluted with homogenizing buffer to equal the final dilution of the sucrose gradient and the synaptosome fractions for western blotting.

For vesicle fractionations, the P2 pellet was resuspended in 10 mL homogenizing buffer and was centrifuged at 12,500 x g for 15 min at 4°C. The supernatant (S2’’) was collected and stored and the pellet (P2’’) was resuspended in 10 mL hypo-osmolar lysis buffer containing 4 mM HEPES pH 7.4 and protease inhibitors (Sigma Protease Inhibitor Cocktail for mammalian cells), and the osmotic lysis was allowed to proceed for 30 min on ice. The lysate was then centrifuged at 25,000 x g for 20 min at 4°C and the supernatant (LS4) was harvested. LS4 was then centrifuged at 245,000 x g for 2 h at 4°C in a swing bucket rotor (SW41). The pelleted vesicle fraction (LP5) was resuspended in 100 μL homogenizing buffer. Fractions were processed for western blotting in 1x SDS as described before. Total protein concentrations of S1, P2’’, and LP5 were estimated using Coomassie blue staining of samples run on acrylamide gels, and concentrations were adjusted accordingly so that the total protein amounts across samples were even.

#### Sample collection and western blotting

Tissues were harvested from mice that were first deeply anesthetized on ice (P0-P5) or with isoflurane chamber (P10-P90). After decapitation, harvested organs were washed in ice-cold PBS, weighed and were homogenized using a glass-Teflon homogenizer in 10% w/v homogenizing buffer (150 mM NaCl, 25 mM HEPES, 4 mM EDTA and 1% Triton X-100, at pH 7.5). Homogenized tissues were incubated for 1 h at 4°C with gentle rotation, then 3x SDS sample buffer was added to a final 1x concentration and denatured by boiling for 10 min at 95°C. Neuronal cultures grown on glass coverslips in 24-well culture plates were harvested in 15 μL 1x SDS sample buffer per coverslip and boiled for 10 min at 95°C. Western blotting was performed according to standard protocols. After SDS-Page electrophoresis, proteins were transferred onto nitrocellulose membranes in a Tris-glycine buffer with 20% methanol for 6.5 h at 4°C and 80 V.

For non-quantitative western blotting using chemiluminescence, nitrocellulose membranes were blocked in TBST (Tris-buffered saline with 0.1% v/v Tween-20) supplemented with 10% (w/v) non-fat milk and 5% (v/v) goat serum for 1 hr at room temperature (RT). Membranes were incubated with primary antibodies in TBST with 5% (w/v) non-fat milk and 2.5% (v/v) goat serum overnight at 4°C. After washing 3 × 10 min with TBST, the membranes were incubated for 1 hr at RT with HRP-conjugated secondary antibodies (S44-S46 in Key Resources Table, 1:10,000, or anti-rat IgG, 1:2,000) in TBST, and washed 3 × 10 min. Membranes were incubated with an enhanced chemiluminescence (ECL) reagent and exposed to X-ray films. For quantitative fluorescent western blotting, nitrocellulose membranes were blocked in Tris-buffered saline (TBS) with 5% (w/v) non-fat milk and 5% (v/v) goat serum for 1 h at RT. Membranes were incubated overnight at 4°C in TBST with 5% w/v BSA with primary antibodies against the protein of interest and an anti-Synapsin-1 antibody as a loading control. After washing 3 × 10 min in TBST at RT, blots were incubated for 1 h in TBST with 5% w/v BSA at RT with fluorescent secondary antibodies (680CW or 800CW conjugated IR dyes: S40-S43 in Key Resources Table, 1:10,000), followed by 3 × 10 min washing in TBST and 3 × 10 min washing in TBS (without Tween-20). Blots were air-dried in the dark and scanned on a LICOR Odyssey Fluorescent Scanner, and the original 16-bit fluorescent images were analyzed in ImageJ software. Each target protein was first normalized to its corresponding Synapsin-1 band to control for loading, then protein levels in each knockout condition were normalized to their corresponding control protein levels. For illustration in figures, images were compressed to 8 bit, resulting in near white background for images with large initial gray value ranges.

The following primary antibodies and concentrations were used in ECL and fluorescent western blotting: mouse anti-β-actin (A127, 1:5,000), rat anti-Clasp2 (A27, 1:500), rabbit anti-Complexin-1/2 (A68), 1:2,000), rabbit anti-ELKS (A141, 1:2,000), rabbit anti-ELKSα (A55, 1:200), rabbit anti-ELKSB (A102, 1:1,000), mouse anti-ELKS1 (A48, 1:200), rabbit anti-ELKS2α (A65, 1:1,000), rabbit anti-ELKS2αB (A143, 1:200), rabbit anti-GFP (A146, 1:2,000), mouse anti-GluA1 (Sysy, 1:500), mouse anti-GM130 (A1, 1:500), mouse anti-HA (A12, 1:500), rabbit anti-Liprin-a3 (A35, 1:2,000), rabbit anti-Munc13–1 (A118, 1:2,000), mouse anti-Neurofilament (A117, 1:500), mouse anti-PSD-95 (A149,1:1,000), rabbit anti-Rab3A (A14, 1:2,000), rabbit anti-Rab6A/B (LifeSpan, 1:500), rabbit anti-Rab6B (A76, 1:500), rabbit anti-RIM1 (A58, 1:500), rabbit anti-SNAP-25 (A109, 1:1,000), mouse anti-Synapsin-1 (A57, 1:1,000), rabbit anti-Synaptobrevin-2 (A135,1:2,000), rabbit anti-Synaptotagmin-1 (A134, 1:500), mouse anti-Synaptophysin-1 (A100, 1:2,000), rabbit anti-Syntaxin-1 (A145, 1:500), rabbit anti-Syntaxin-6 (A186, 1:1,000), mouse anti-T7 (A49, 1:2,000), rabbit anti-VAMP4 (Sysy, 1:400), and rabbit anti-VCP (A33, 1:1,000). For further information on primary antibodies, see Key Resources Table.

#### Real time quantitative PCR

Real-time quantitative PCR (RT-qPCR) analysis of mRNA levels was performed as previously described (Liu et al., 2014). To measure Rab6A and Rab6B expression, RNA was isolated from cultured hippocampal neurons of Rab6B^+/+^ and Rab6B^−/−^ littermates using an RNA extraction and stabilizing buffer (iScript RT-qPCR Sample Preparation Reagent, Bio-Rad). Cultures were rinsed with PBS and incubated with 50 μL of extraction buffer for 30 s, followed by centrifugation for 1 min at 13,000 x g to pellet cell debris. 1 μL of supernatant was used in a 10 μL qPCR reaction, each sample was run in three replicates, and samples were collected from three independent batches of cultures. Probe-based one-step RT-qPCR was performed following standard procedures and fluorescent signal amplification was quantified by spectrophotometry, using TaqMan Gene Expression Assays (Thermo Fisher) and the iScript Reverse Transcriptase (Bio-Rad). The following TaqMan assays were used: Rab6A (assay ID: Mm00445868_m1, gene name *Rab6A*), Rab6B (assay ID: Mm00620652_m1, gene name *Rab6B*), Synapsin-1 (assay ID: Mm00449772_m1, gene name: *Syn1*). Data were analyzed by determining the cycle threshold values (CT) relative to the corresponding Synapsin-1 mRNA levels. Relative expression ratios were expressed as 2^−∆∆C^_T_, where ∆∆C_T_ = ∆C_T Rab6_^−/−^ - ∆C_T Rab6_^+/+^, and ∆CT is the Synapsin-1 normalized value.

#### Immunofluorescent staining and confocal microscopy

Neurons grown on glass coverslips were washed twice with warm PBS and fixed in ice-cold 4% paraformaldehyde in PBS for 15 minutes at RT, permeabilized in blocking solution with 0.1% Triton X-100, 3% BSA in PBS (TBP) for 45 min at RT, incubated with primary antibodies in blocking solution overnight at 4°C, followed by 3 × 10 min washes in TBP at RT. Secondary antibodies conjugated to Alexa Fluor 488, 546, or 633 were used for detection (S4, S5, S15, S16, S32, S33, S34, 1:500) after overnight incubation at 4°C, followed by 3 × 10 min washes in TBP at RT. Air-dried coverslips were mounted on to glass slides in Fluoromount-G mounting medium. Slides were allowed to dry for two days at RT in the dark before they were imaged or stored at 4°C. Confocal images were acquired on Olympus FV1000 or FV1200 microscopes with 100x or 60x oil immersion objectives (1.4 N.A.), using the same acquisition settings for all samples within an experiment. Single confocal sections were analyzed with ImageJ as described before (Liu et al., 2014). For quantification of synaptic protein levels in confocal images, regions of interests (ROIs) were defined by thresholded binary Bassoon or Synaptophysin-1 objects and signal intensities of the protein of interest were quantified within these objects on raw, unadjusted images. For representative images in figures, image areas were selected visually and rotated to display them in a similar orientation followed by smoothening and cropping at higher pixel density (300 dpi). All adjustments were made identically for each condition within an experiment. The experimenter was blind to the experimental condition for all data acquisition and analyses.

The following primary antibodies and concentrations were used for experiments solely performed with confocal microscopy: guinea pig anti-Bassoon (A67, 1:500), mouse anti-ELKS1 (A48, 1:200), mouse anti-GFP (A34, 1:1,000), rabbit anti-GFP (A146, 1:2,000), mouse anti-GM130 (A1, 1:500), mouse anti-HA (A12, 1:500), mouse anti-Map2 (A108, 1:500), rabbit anti-Rab6A/B (LifeSpan, 1:500), rabbit anti-RFP (A81, 1:1,000), and guinea pig anti-Synaptophysin-1 (A106, 1:1,000). For further information on primary antibodies, see Key Resources Table.

#### STED microscopy and analyses

STED microscopy was performed as described before (de Jong et al., 2018; Wong et al., 2018). Neurons were grown on 0.15-mm-thick glass coverslips and were processed and stained with primary antibodies as described for confocal microscopy above. Secondary antibodies conjugated to Oregon green 488, Alexa Fluor 555, and Alexa Fluor 633 were used for detection (S10, S11, S18, S22, S23, S32, S33, S34, 1:500 for confocal channel, 1:200 for STED channels). STED imaging was performed on a Leica SP8 Confocal/STED 3X microscope using a 100x oil immersion objective (1.44 N.A.). Alexa Fluor 633, Alexa Fluor 555 and Oregon green 488 were excited with 633 nm, 555 nm and 488 nm white light lasers respectively (1–6% of 1.5 mW laser power) in this order. During STED scanning, Alexa Fluor 555 signals were depleted with a 660 nm laser (50% of max power), and Oregon Green 488 signals were depleted with a 592 nm laser (75% of max power). Both depletion lasers were time-gated, and were set to 30% z depletion. Two-times line accumulation and two-times frame averaging were applied during STED scanning. STED images were acquired at a pixel size of 22.7 × 22.7 nm^2^ in Figure 3 and with 14.2 × 14.2 nm^2^ in all other STED experiments. In all STED experiments, the synapse marker (Synapsin-1 or Synpatophysin-1) was acquired in a confocal channel at 633 nm excitation. Identical settings were applied to all samples within an experiment. All image analyses were performed in ImageJ. For analysis of subsynaptic intensity distribution (Figures 2G–2H, S2E, S2F, 3E, 3G, 3H, 3J, S3J, 5G, and 5H), line-scan intensity profiles of side-view synapses were obtained for the synaptic vesicle cluster marker (either Synapsin-1 or Synaptophysin-1, imaged with confocal microscopy), the active zone or PSD marker (either Bassoon or PSD-95, imaged by STED), and the test protein (imaged by STED). The work flow of side-view synapse selection and line profile analysis is outlined in Figure S2D. In brief, side-view synapses were selected manually from raw, unprocessed images based on synaptic vesicle markers and the active zone or PSD marker that appeared as a bar on one side of the synapse. All synapses were selected blind to the condition and to the target protein signal. A rectangular 0.2 μm x 1.0 μm bar was placed perpendicular through the center of the active zone or PSD signal. Intensity values within this bar were extracted using a custom ImageJ program for the active zone or PSD marker and for the target proteins. The intensity values (using a rolling average of three consecutive values for each data point) of the target proteins were plotted relative to the active zone or PSD peaks aligned to zero. All quantitative analyses were performed on original images without adjustments and were done identically for all experimental conditions. For representative STED images of individual synapses in figures, synapses were selected visually and rotated to display them in a similar orientation followed by smoothening and cropping at higher pixel density (300 dpi). All adjustments were made identically for each condition within an experiment. For analyses of synapses irrespective of the viewing angle (Figures 1H–1K, 4H, 4I, 5E, 5F, S6A, and S6B), the confocal signal of the synapse marker (either Synapsin-1 or Synaptophysin-1) of individual synapses was used to create ROIs after thresholding. Intensities of target proteins within these ROIs, acquired by STED, were then measured. To determine the non-active zone fraction of ELKS (Figure 3K), a second ROI was generated for Bassoon that colocalized with Synapsin-1 (Bassoon+Synapsin objects). ELKS1 or ELKS2 intensities (acquired by STED) were then measured both within the Synapsin ROIs and the Bassoon+Synapsin ROIs. To determine the fraction of ELKS signal that fell outside the active zone, we calculated (1 – ELKS-_Synapsin+Bassoon-ROIs_/ELKS_Synapsin-ROIs_). For correlation analyses of endogenous signals in Figures 1K and S6B, 1.5 × 1.5 μm^2^ images were selected around single synapses, and the total signal intensities of Rab6B, ELKS1, or Synaptophysin-1 were measured (without background subtraction). For correlation of overexpressed Rab6A or Rab6B intensities with ELKS1 in Figure 6, thresholded Synaptophysin-1 objects were used for synapse selection and intensity measurements. For all image acquisition and analyses the experimenter was blind to the experimental condition.

The following primary antibodies and concentrations were used: guinea pig anti-Bassoon (A67, 1:500), mouse anti-ELKS1 (A48, 1:200), rabbit anti-ELKS2 (A136, 1:200), mouse anti-PSD-95 (A149,1:1,000), rabbit anti-Rab6B (A76, 1:500), rabbit anti-RIM1 (A58, 1:500), guinea pig anti-Synaptophysin-1 (A106, 1:1,000), rabbit anti-Synaptophysin-1 (A64, 1:1,000), mouse anti-Synapsin-1 (A57, 1:1,000), and rabbit anti-Synapsin-1 (A99, 1:1,000). For further information on primary antibodies, see Key Resources Table.

Bassoon was used as a marker protein in for STED or confocal microscopy. In confocal microscopy, the synaptic vesicle and active zone markers cannot be separated due to the limited resolution, and Bassoon was used as a marker of synapses in Figures 1D, S3H, S3I, 7E, and S7A. In STED microscopy, the subsynaptic distribution of proteins is possible and Bassoon was used as an active zone marker in Figures S2E, S2F, 3E, and 3H.

#### Electron microscopy

Electron microscopy was performed as previously described (Wang et al., 2016; Wong et al., 2018). For fixation by high-pressure freezing, neurons cultured on 6 mm carbon-coated sapphire coverslips were frozen using an Leica EM ICE high-pressure freezer in extracellular solution (140 mM NaCl, 5 mM KCl, 2 mM Ca^2+^, 2 mM Mg^2+^, 10 mM HEPES pH 7.4, 10 mM glucose with ~310 mOsm, 50 μM picrotoxin, 50 μM AP5, and 20 μM CNQX). High-pressure frozen samples were freeze-substituted (1% glutaraldehyde, 1% osmium tetroxide, 1% water and anhydrous acetone), Epon infiltrated, and polymerized by baking at 60°C for 2–3 days, then at 100°C overnight immediately before ultrathin sectioning at 50 nm. To enhance contrast, mounted sections were stained for 10 s with lead citrate. For chemical fixation, neurons on standard glass coverslips were fixed with 2% glutaraldehyde in 0.1 M sodium cacodylate buffer for 10 min at 37°C, followed by processing by the Electron Microscopy Facility at Harvard Medical School. Samples were stained in a 1% osmium tetroxide/1.5% potassium ferrocyanide buffer for 1 h at RT, washed once in water and three times in maleate buffer (pH 5.15), stained with 1% uranyl acetate for 1 h, dehydrated in of EtOH and propylene oxide, resin infiltrated, and baked for 24 h at 60°C before sectioning at 50 nm. Sections of high-pressure frozen and glutaraldehyde fixed samples were imaged on a transmission electron microscope (JEOL 1200 EX at 80 kV accelerating voltage) at 15,000 x for axonal segments and 30,000 x for boutons and were processed with ImageJ. Bouton analyses including area, the total number of vesicles, the PSD length, and the number of docked vesicles at the active zone was done using SynapseEM, a MATLAB program provided by Drs. Broeke and Verhage. A vesicle was considered docked if there was no visible white space between its membrane and the presynaptic plasma membrane opposed to the PSD. Endosomes were defined as intracellular membrane-enclosed structures that are larger than 50 nm in diameter and have a clear lumen. Axon identification was set up in trial experiments by describing morphology of axons connected to presynaptic boutons, and were later chosen for analysis based on the following criteria: plasma membranes were parallel and non-tapering, at least a 1 μm segment had to be within the field of view, the width had to be within 0.2 μm and 0.75 μm, and parallel microtubules were present throughout the whole segment. Axonal vesicles were defined as vesicles with diameters of 50 nm or less residing in axonal segments away from boutons. When a bouton was present on an axon, a vesicle (measured from its center) had to be at least 150 nm away from the center of any vesicle residing within the presynaptic bouton. A group of a minimum 20 vesicles was considered to be a bouton, where the center of any vesicle was at most 100 nm away from the center of its nearest neighbor. The experimenter was blind to the experimental conditions during all data acquisition and analyses.

#### Protein expression and purification

GST- and His-tagged fusion proteins were expressed and purified according to standard procedures. In brief, proteins were expressed at 20°C in electrocompetent *E. coli* BL21 cells after induction with 0.05 mM isopropyl β-D-1-thiogalactopyranoside (IPTG) for 20 h, and pelleted by centrifugation (45 min on 3,500 x g at 4°C). For purification of GST-fusion proteins, bacterial pellets were resuspended in GST-lysis buffer (1x PBS, 0.5 mg/mL lysozyme, 0.5 mM EDTA, 1 μM PMSF, 1 μM Bestatin, 1 μM Pepstatin A, and 5 μM E-64, pH 8.0) and lysed for 30 min on ice, then briefly sonicated using an ultrasonic cell disruptor (Branson Sonifier 450), and pelleted by centrifugation (45 min on 11,200 x g at 4°C) with a JA-20 rotor. Cleared bacterial supernatants were incubated with glutathione-Sepharose resin (GE Healthcare) for 1.5 h at 4°C with gentle rotation, then washed three times in 10-fold volume ice-cold PBS (1 min centrifugation at 500 x g, 4°C), and were stored in 5 to 10-fold volume PBS at 4°C and used within 5 days of purification. The concentration of purified GST-fusion proteins was determined by comparing them against known BSA concentrations using SDS-gel electrophoresis and Coomassie staining. The following GST-tagged proteins were produced from pGEX-KG2 constructs: GST alone (pAJ13017), Rab3A Q81L (pHN150605), Rab3A T36N (pHN150606), Rab6A Q72L (pHN150809), Rab6A T27N (pHN150808), Rab6B Q72L (pHN160708), Rab6B T27N (pHN160709), ELKS1αB 2-208 (pLB12022), ELKS1αB 209-358 (pLB12023), ELKS1αB 359-696 (pLB12024), ELKS1αB 697-992 (pLB12025), ELKS1αB 654-955 (pHN160636), ELKS1αB 654-971 (pHN160637), ELKS1αB 654-992 (pHN160638), ELKS1αB 769-992 (pHN160615), ELKS1αB 808-992 (pHN160618), ELKS1αB 850-992 (pHN160619), ELKS1αB 808-971 (pHN160617), ELKS2αB 765-884 (pHN160912). For purification of His-tagged fusion proteins, bacterial pellets were resuspended in His-lysis buffer (300 mM NaCl, 10 mM imidazole, 50 mM NaH2PO4, 0.5 mg/mL lysozyme, 1 μM PMSF, 1 μM Bestatin, 1 μM Pepstatin A, and 5 μM E-64, pH 8.0) and lysed for 30 min on ice, then briefly sonicated using an ultrasonic cell disruptor (Branson Sonifier 450), and pelleted by centrifugation (45 min on 11,200 x g at 4°C). The cleared bacterial supernatant was incubated with Ni-NTA agarose (Thermo Fisher) for 1 hr at 4°C with gentle rotation and His-tagged proteins were eluted from the resin on a column via gravity flow in the same buffer containing increasing amounts of imidazole (1 mL 20 mM as pre-wash, 1.5 mL 100 mM, 2 × 2 mL 220 mM, and 1 mL 20 mM imidazole buffer as post-wash). Protein concentration in each fraction was determined by SDS-PAGE electrophoresis and Coomassie stain relative to known BSA concentrations. Most protein was detected in the second and third fractions with 200 mM imidazole and essentially no eluent was detected in the pre- and post-wash fractions. The second and third fractions were pooled and dialyzed overnight at 4°C into a buffer containing 150 mM NaCl and 25 mM HEPES (pH 8.0), and protein concentrations after dialysis were estimated by comparing them to known BSA concentrations using SDS-gel electrophoresis followed by Coomassie staining. Protein aliquots were stored at −80°C until use. The following His-tagged recombinant proteins were produced from pET28a constructs: Rab6A (pHN160210), Rab6A Q72L (pHN160211), Rab6A T27N (pHN160212), Rab6B (pHN160701), RAB6B Q72L (pHN160702), RAB6B T27N (pHN160703).

#### GST affinity purifications from mouse brain lysates and HEK293T cells

For affinity purifications of endogenous proteins or overexpressed proteins, lysates were prepared from brains of 6-week old mice (one brain/10 ml) or from HEK293T cells, respectively. For prey protein expression, HEK293T cells were transfected with pCMV or lentiviral vectors (pFSW) expressing HA-ELKS1αA (pLB12010), HA-ELKS1αB (pLB12011, pHN161031), HA-ELKS1βB (pLB12013), HA-ELKS2βB (pLB14074) or HA-ELKS1^∆955-971^ (pHN170936) using 20 μg DNA per T75 flask via standard Ca^2+^-phosphate transfection protocol, and cells were harvested 24 h after transfection. HEK293T cells were washed in ice-cold PBS, and collected in homogenizing buffer containing 100 mM NaCl, 4 mM EGTA, 25 mM HEPES (pH 7.4), 1 mM DTT, 1% w/v Triton X-100 and 1X Sigma Protease Inhibitor Cocktail for mammalian cells. Mouse brains (in the same homogenizing buffer) or HEK293T cells were homogenized with 3 × 10 strokes on ice using a glass-Teflon homogenizer, solubilized with gentle rotation at 4°C for 1 h, followed by centrifugation at 118,000 x g for 1 h at 4°C. The supernatant was precleared from glutathione-binding proteins by incubating lysates with 200 μL of a 50% slurry of glutathione-Sepharose beads for 30 min at 4°C with gentle rotation. Subsequently, 10 μg of GST-Rab proteins (active and inactive mutants) purified as described above were added to 0.5 mL cleared cell lysate (either brain or HEK293T). All assays were equilibrated with beads to contain a final glutathione-Sepharose bead-volume of 20 ml. The binding reaction was supplemented with 6 mM Mg^2+^ and incubated for 1.5 h at 4°C with gentle rotation. Beads were washed 6 times with 1.5 mL homogenizing buffer (protease inhibitors were not included in the washes) at 4°C, and proteins were eluted from the beads with 75 μL 1 x SDS sample buffer and processed for western blotting as described in previous sections, loading 15 μL of eluted bead sample on the SDS gels. For control, input solutions were loaded at 5% of the total input in the binding reaction. Unless otherwise noted, at least three independent affinity purifications were performed for each condition, and one representative experiment is shown in the figures.

#### Recombinant GST affinity purifications

Recombinant affinity purification assays were used to assess direct binding between GST-ELKS fragments immobilized on glutathione-Sepharose beads and soluble His-Rab6 proteins. For recombinant affinity assays with active and inactive point mutant Rabs, concentrated His-tagged Rab protein stocks (described above) were diluted to 1.2 μM in a binding buffer containing 150 mM NaCl, 20 mM HEPES (pH 7.4), 4 mM EGTA, 1 mM DTT, 1% w/v Triton X-100, and 0.1 mg/ml BSA. Solutions containing the His-tagged proteins were precleared by incubating lysates with 200 μL of a 50% slurry of glutathione-Sepharose beads for 30 min at 4°C with gentle rotation and centrifuged with 25,000 x g for 10 min at 4°C. For the assay, 0.5 mL of the precleared supernatant supplemented with 5 mM Mg^2+^ was incubated with GST-protein beads (20 mL final glutathione-Sepharose bead-volume in all assays) for 1 h at 4°C with gentle rotation. The molar ratio of GST-ELKS and His-Rab proteins was 1:4 (0.3 mM GST-ELKS and 1.2 mM of His-Rab input) in assays where the His-Rab point mutants were used as input. After the assay, glutathione beads were washed 6 times with 1.5 mL in the same buffer at 4°C, and proteins were eluted from the beads with 75 ul 1 x SDS sample buffer and processed for western blotting as described above, loading 15 μL of eluted bead sample on SDS-PAGE gels. For control, input solutions were loaded at 5% of total input in the binding reaction. His-Rab6A inputs containing a T7 tag immediately after the N-terminal His-tag were visualized by a T7 antibody (A49, 1:2,000). For recombinant affinity assays with GTP analogs, non-hydrolysable forms of GTP (GMP-PNP) or GDP were added. His-tagged Rab protein solutions were first coupled with the non-hydrolysable GMP-PNP or GDP by diluting the protein stocks to 1.2 μM final concentration in a coupling buffer without Mg^2+^ (150 mM NaCl, 20 mM HEPES pH 7.4, 1 mM DTT, 1% w/v Triton X-100, 0.1 mg/mL BSA, 10 mM EDTA, 0.1 mM GMP-PNP or GDP). The coupling was quenched after 1 h at 4°C by supplementing the protein solution with 20 mM Mg^2+^. Coupled Rab protein solutions were precleared and the experiments were performed as described above.

#### Live imaging of neuronal cultures

For live imaging, wild-type neuronal cultures were transfected with Cerulean-ELKS1αB (pMYW12018) or Cerulean-Rab6B (pHN160704), or ELKS1α/β control and cKO neurons were transfected with Cerulean-Rab6B (pHN160704) in pFSW vectors in which expression is driven by a human Synapsin promoter. Cell cultures were transfected at DIV12 using Ca^2+^-phosphate, and were co-transfected with tdTomato-SV2A (pHN141024). For transfection, the culture medium was removed and stored for the duration of the procedure, and the neurons were washed 3 × 10 min in warm MEM. 0.5 mL of warm MEM per well supplemented with 50 μM AP5 to reduce excitotoxicity were added to each well for 10 min before transfection. Cultures were transfected with a total of 4 μg of DNA in 150 μL transfection solution per well: 3 μg ELKS1αB + 1 μg SV2A or 2 μg Rab6B + 2 μg SV2A. The DNA/Ca^2+^/HEPES solution was allowed to incubate for 5 min before 150 μL solution was added to each well. Precipitate formation was monitored under a light microscope where precipitates started appearing as small pebbles. Once the pebbles started aggregating (after approximately 10 min at RT), the transfection solution was immediately removed and cells were washed twice in 1 mL AP5-supplemented MEM. Cells were then washed 2 × 10 min in AP5/MEM and 3 × 10 min in MEM alone. Between each wash, cells were placed back in the tissue culture incubator. After the final wash, the original cell culture medium was added back onto cells. Cultures were monitored for overall health and were imaged 2–3 days post-transfection. Live imaging was carried out on an Olympus light microscope with an LED light source (*p*E-4000). The following single-band filter sets were used: Brightline® CFP (Semrock) for Cerulean and ET-Cy3 (Chroma) for tdTomato. Images were recorded using a sCMOS camera (Hamamatsu ORCA-Flash4.0). Live cultures were imaged at 35°C in Hybernate-A (BrainBits) solution. Cerulean was excited at 435 nm and tdTomato was excited at 550 nm, sequentially. Time-lapse images for Cerulean signals were recorded for 2 min with 1 frame/s imaging speed, and slight focus adjustments were made manually if necessary. Still images of tdTomato (SV2A) signal were captured immediately before and after time-lapse imaging of the Cerulean signal. Axons were identified visually by their bead-like morphology, and raw images were analyzed in ImageJ. The position of SV2A stationary puncta was determined by the average of the pre- and post-time-lapse tdTomato signals. The two SV2A images were thresholded to the signal intensity in the first image and a binary image was created. Only SV2A puncta that were present in both images would fall above thresholding limits and were used to mark synapses. Kymographs were generated from a three-pixel wide line-scan of the Cerulean signals along axons, where x axis shows distance and y axis shows elapsed time in frames. In these kymographs, moving objects appear as diagonal lines, while stationary objects appear as straight vertical bars. An object was considered stationary if it appeared as a straight vertical bar on the kymograph for the entire 2-min duration. To assess the fraction of stationary Cerulean objects (ELKS1 or Rab6B) that accumulated at SV2A-tdTomato puncta, thresholded binary objects were created from the Cerulean time-lapse and their association with the tdTomato objects (from the binary image described above) was measured. A stationary Cerulean object was considered to be synaptic if it had > 0% overlap with a tdTomato object. For analysis of movement, the path of an event with diagonal segments on the kymograph for the entire 2-min duration was manually outlined including pauses. If a moving object paused, segments between pauses were not counted as independent events if the path of movement could be determined unambiguously. One outlined path (including pauses and segments between pauses) equaled to one movement event. To assess flux, the total number of moving paths per kymograph was normalized to the length of the axon and normalized to the imaging time. To assess instant speed, a continuous line segment was drawn between pauses and the speed was calculated from the x (mm) and y (s) components of the diagonal line. To asses net speed, a continuous line segment was drawn between the point or appearance and point of disappearance including all pauses, and the speed was calculated as described above. For representative images in Figs. 6A and 6E, axon segments were selected visually, rotated, smoothened and cropped at higher pixel density (300 dpi). The representative kymographs of a different set of axons in Figures 6C and 6G were generated, smoothened and cropped at high pixel densities (300 dpi) for display. All adjustments were made identically for each condition within an experiment. All experiments and analyses were performed by an experimenter blind to the genotype.

#### Experiments with mistargeting of ELKS or Rab6 to mitochondria

To localize ELKS1 or Rab6 to the mitochondrial membrane, a mito-tag composed of the trans-membrane domain of the mitochondrial Tom20 protein (Kanaji et al., 2000) and a Cerulean fluorescent protein was fused onto their N terminus. In the experiments with mitochondrially localized ELKS, wild-type hippocampal neurons were infected at DIV3 with 200 μL lentiviral solution expressing mito-ELKS1αB (pHN161033) or mito-ELKS1^∆955-971^ (pHN190429), or mito-tag alone (pHN161037, 50 μL lentiviral at DIV5 as the small mito-Cerulean expressed much more efficiently). Expression levels were monitored via western blotting with anti-GFP antibodies that recognize Cerulean. For electron microscopy, high-pressure frozen cultures were processed and imaged as described above. The number of small vesicles (diameter ≤ 50 nm) associated with the mitochondrial surface was counted. A vesicle was considered tethered if it was within 70 nm from a mitochondrial surface. The maximum distance of 70 nm was determined by measuring the longest visible tether between vesicles and mitochondria that were sometimes present in these experiments; however, a tether did not have to be visible as long as the vesicle fell within the 70-nm-distance from the mitochondrial membrane. The total number of vesicles counted around a mitochondrion was normalized to the mitochondrion perimeter (μm), and Figure 7C shows the number of vesicles normalized to 3-μm mitochondrial perimeter, which was the average mitochondrial perimeter. In the experiments with mito-Rab6, wild-type hippocampal cultures were infected at DIV3 with 150 μL lentivirus HA-ELKS1αB (pHN161031) or HA-ELKS1^−∆^955-971 (pHN170936). Subsequently, the same cultures were infected at DIV5 with 100 μL mito-Rab6B_QL_ (pHN181203) or mito-Rab6B_TN_ (pHN181203). Cultured neurons were processed for confocal imaging or western blotting as described before. Neurons were immunostained with anti-GFP, anti-HA and anti-Bassoon antibodies and images were acquired by confocal microscopy, and Bassoon was used as a synapse marker in these experiments. Images were analyzed in ImageJ, using the BioVoxxel plugin. To quantify the fraction of Bassoon objects that contained mito-Rab6, we constructed histograms of the overlap of Bassoon and Rab6. To plot the data, we set an overlap cutoff such that at least 50% of the Bassoon area had to contain mito-Rab6 to be considered Rab6-positive. To assess whether the mito-tag localized proteins to the mitochondrial surface, mito-Cerulean-ELKS1αB localization was compared with Cerulean-ELKS1αB in neurons that simultaneously expressed the mitoDsRed mitochondrial marker (pHN161038, 50 μL mito-DsRed lentivirus was used to transduce neurons at DIV7) in the case of ELKS. Neurons were immunostained with anti-GFP antibodies (A34, 1:1000) and anti-RFP antibodies (A81, 1:1000) to detect Cerulean and DsRed, respectively, co-stained with anti-Synaptophysin-1 antibodies to mark synapses. All mitochondrial targeting experiments and analyses were performed by an experimenter blind to the genotype.

### QUANTIFICATION AND STATISTICAL ANALYSIS

Unless otherwise noted, all data are means ± SEM and p values are shown as * p < 0.05, ** p < 0.01, *** p < 0.001. The following experiments were analyzed by Pearson’s correlation to determine the linear relationship between fluorescent signal intensities acquired either by confocal or STED superresolution imaging: Figures 1J, 1K,6K, S6A, S6B, and S6E. The Mendelian survival ratios of mouse lines were analyzed by Chi-square test in the following experiments: Figures S1E, S4A, and S4B. Statistical comparisons between more than two conditions were done by one-way ANOVA followed by Holm-Sidak’s multiple comparisons test to one control condition as indicated in each figure in the following experiments: Figures 5B, 5D, 5F, S5B, 7C, and 7F. Statistical comparisons of the line-scan profiles of STED side-view synapses were done using 2-way ANOVA, followed by Holm-Sidak’s multiple comparisons test as indicated in each figure in the following experiments: Figures 2H, S2F, 3J, 5H, S5C (all on a 200 nm-wide area centered around the peak fluorescence), 3G (on 100 nm to the right and 300 nm to the left of its own peak), S3J (on 500 nm toward the left of the Bassoon peak). All other experiments were analyzed by Student’s t tests. Statistical analyses and data graphing for illustration were done using GraphPad Prism. Unless otherwise noted, all experiments were done using a minimum of three independent cultures and, in each culture, multiple cells or multiple images were analyzed. All images were processed and analyzed in ImageJ, unless otherwise noted. The experimenter was blind to conditions throughout data acquisition and analyses.

## Supplementary Material

1

2

## Figures and Tables

**Table:** KEY RESOURCES TABLE

REAGENT or RESOURCE	SOURCE	IDENTIFIER
Antibodies		

guinea pig anti-Bassoon (A67)	Sysy	RRID:AB_2290619
mouse anti-β-actin (A127)	Sigma	RRID:AB_476692
rat anti-Clasp2 (A27)	Absea	KT68
rabbit anti-Complexin-1/2 (A68)	Dr. T.C. Sudhof	P942
mouse anti-ELKS1 (A48)	SCBT	RRID:AB_10841908
rabbit anti-ELKS2 (A136)	Kaeser laboratory	HM1029
rabbit anti-ELKS2α (A65)	Dr. T.C. Südhof	U5004
rabbit anti-ELKS2αB (abErc2) (A143)	Abcam	RRID:AB_731499
mouse anti-ELKSα (ELKS-30) (A55)	Abcam	RRID:AB_869944
rabbit anti-ELKSB (A102)	Dr. T.C. Südhof	4790
rabbit anti-ELKS (A141)	Kaeser laboratory	HM1083
mouse anti-GFP (JL8) (A34)	CloneTech	RRID:AB_2313808
rabbit anti-GFP (A146)	Dr. T.C. Südhof	RRID:AB_2636878
mouse anti-GluA1 (GluR1)	Sysy	RRID:AB_2113443
mouse anti-GM130 (A1)	BD Bioscience	RRID:AB_398141
mouse anti-HA (A12)	Biolegend	RRID:AB_2565006
rabbit anti-Liprin-a3 (A35)	Dr. T.C. Südhof	RRID:AB_2617056
mouse anti-Map2 (A108)	Sigma	RRID:AB_477193
rabbit anti-Munc13-1 (A118)	Dr. N. Brose	n/a
mouse anti-Neurofilament (SMI312R) (A117)	Covance	RRID:AB_2315329
mouse anti-PSD-95 (A149)	NeuroMab	RRID:AB_10698024
rabbit anti-Rab3A (A14)	Dr. T.C. Südhof	T957
rabbit anti-Rab6A/B	LifeSpan	LS-B10800
rabbit anti-Rab6B (A76)	LifeSpan	LSC379639
rabbit anti-RIM1 (A58)	Sysy	RRID:AB_887774
rabbit anti-RFP (A81)	Rockland	RRID:AB_2209751
rabbit anti-SNAP-25 (A109)	Sysy	RRID:AB_887790
mouse anti-Synapsin-1 (A57)	Sysy	RRID:AB_2617071
rabbit anti-Synapsin-1 (A99)	Abcam	RRID:AB_2200097
rabbit anti-Synaptobrevin-2 (A135)	Sysy	RRID:AB_887810
guinea pig anti-Synaptophysin-1 (A106)	Sysy	RRID:AB_1210382
mouse anti-Synaptophysin-1 (A100)	Sysy	RRID:AB_887824
rabbit anti-Synaptophysin-1 (A64)	Sysy	RRID:AB_887905
rabbit anti-Synaptotagmin-1 (A134)	DSHB	RRID:AB_2199314
rabbit anti-Syntaxin-1 (A145)	DSHB	RRID:AB_528484
rabbit anti-Syntaxin-6 (A186)	Sysy	RRID:AB_887854
mouse anti-T7 (A49)	Novagen	RRID:AB_10807769
rabbit anti-VAMP4	Sysy	RRID:AB_887816
rabbit anti-VCP (A33)	Dr. T.C. Sudhofü	K330
goat anti-mouse Oregon Green 488 IgG (S10)	Thermo Fisher	RRID:AB_2534088
goat anti-mouse Alexa Fluor 488 IgG (S4)	Thermo Fisher	RRID:AB_2534088
goat anti-mouse Alexa Fluor 546 IgG (S15)	Thermo Fisher	RRID:AB_2534071
goat anti-mouse Alexa Fluor 555 IgG (S18)	Thermo Fisher	RRID:AB_141822
goat anti-mouse Alexa Fluor 633 IgG (S32)	Thermo Fisher	RRID:AB_2535718
goat anti-rabbit Oregon Green 488 IgG (S11)	Thermo Fisher	RRID:AB_2539798
goat anti-rabbit Alexa Fluor 488 IgG (S5)	Thermo Fisher	RRID:AB_2576217
goat anti-rabbit Alexa Fluor 546 IgG (S16)	Thermo Fisher	RRID:AB_2534093
goat anti-rabbit Alexa Fluor 555 IgG (S22)	Thermo Fisher	RRID:AB_2535849
goat anti-rabbit Alexa Fluor 633 IgG (S33)	Thermo Fisher	RRID:AB_2535718
goat anti-guinea pig Alexa Fluor 555 IgG (S23)	Thermo Fisher	RRID:AB_2535856
goat anti-guinea pig Alexa Fluor 633 IgG (S34)	Thermo Fisher	RRID:AB_2535757
donkey anti-mouse IRDye 680RD IgG (S40)	LI-COR	RRID:AB_109536 28
donkey anti-mouse IRDye 800CW IgG (S42)	LI-COR	RRID:AB_621847
donkey anti-rabbit IgG IRDye 680RD IgG (S41)	LI-COR	RRID:AB_10954442
donkey anti-rabbit IgG IRDye 800CW IgG (S43)	LI-COR	RRID:AB_621848
goat anti-mouse peroxidase-conjugated (S44)	MP Biologicals	RRID:AB_2334540
goat anti-rabbit peroxidase-conjugated (S45)	MP Biologicals	RRID:AB_2334589
goat anti-rat peroxidase-conjugated (S46)	Abcam	RRID:AB_10680316

Experimental Models: Organisms/Strains		

Mouse: C57BL/6N-*Rab6b*^*em1(IMPC)J*^/Mmucd	Jackson Laboratory	JAX Stock# 028993 RRID:MMRRC_049340-UCD
Mouse: C57BL/6N-*A*^*tm1Brd*^ *Erc1*^*tm1a(EUCOMM)Hmgu*^/BayMmucd	KOMP2 BaSH Consortium Group/MMRRC at UC Davis	RRID:MMRRC_041523-UCD
Mouse: STOCK *Erc1*^*tm2.1Sud*^/J	Liu et al., 2014	RRID:IMSR_JAX:015830
Mouse: STOCK *Erc2*^*tm1.2Sud*^/J	Kaeser et al., 2009	RRID:IMSR_JAX:015831
Mouse: STOCK *Rims1*^*tm3Sud*^/J	Kaeser et al., 2008	RRID:IMSR_JAX: 015832

Recombinant DNA		

pFSW EGFP ∆cre	Liu et al., 2014	pHN131014
pFSW EGFP cre	Liu et al., 2014	pHN131015
pET Rab6A	This paper	pHN160210
pET Rab6A Q72L	This paper	pHN160211
pET Rab6A T27N	This paper	pHN160212
pGEX Rab6A Q72L	This paper	pHN150809
pGEX Rab6A T27N	This paper	pHN150808
pFSW Cerulean Rab6A Q72L	This paper	pHN160326
pET Rab6B	This paper	pHN160701
pET Rab6B Q72L	This paper	pHN160702
pET Rab6B T27N	This paper	pHN160703
pGEX Rab6B Q72L	This paper	pHN160708
pGEX Rab6B T27N	This paper	pHN160709
pFSW Cerulean Rab6B	This paper	pHN160704
pFSW Cerulean Rab6B Q72L	This paper	pHN160705
pFSW Cerulean Rab6B T27N	This paper	pHN160706
pFSW Tom20TMD Cerulean Rab6B Q72L	This paper	pHN181203
pFSW Tom20TMD Cerulean Rab6B T27N	This paper	pHN181204
pFSW Tom20TMD Cerulean ELKS1αB	This paper	pHN161033
pFSW Tom20TMD Cerulean ELKS1αB ∆955-971	This paper	pHN190429
pFSW Tom20TMD Cerulean	This paper	pHN161037
pFSW Cerulean ELKS1αB	This paper	pMYW12018
pFSW HA ELKS1αB	This paper	pHN161031
pFSW HA ELKS1αB D955-971	This paper	pHN170936
pCMV HA ELKS1αA	This paper	pLB12010
pCMV HA ELKS1αB	This paper	pLB12011
pCMV HA ELKS1βB	This paper	pLB12013
pCMV HA ELKS2αB	This paper	pLB14065
pCMV HA ELKS2βB	This paper	pLB14074
pGEX ELKS1αB 2-208	This paper	pLB12022
pGEX ELKS1αB 209-358	This paper	pLB12023
pGEX ELKS1αB 359-696	This paper	pLB12024
pGEX ELKS1αB 697-992	This paper	pLB12025
pGEX ELKS1αB 654-955	This paper	pHN160636
pGEX ELKS1αB 654-971	This paper	pHN160637
pGEX ELKS1αB 654-992	This paper	pHN160638
pGEX ELKS1αB 769-992	This paper	pHN160615
pGEX ELKS1αB 808-992	This paper	pHN160618
pGEX ELKS1αB 850-992	This paper	pHN160619
pGEX ELKS1αB 808-971	This paper	pHN160617
pGEX ELKS2αB 765-884	This paper	pHN160912
pFSW mitoDsRed	This paper	pHN161038
pFSW tdTomato SV2A	This paper	pHN141024

Software and Algorithms		

Fiji/ImageJ	NIH	RRID: SCR_002285
GraphPad Prism	GraphPad	RRID: SCR_002798
Lasergene Core Suite	DNASTAR	RRID: SCR_000291
MATLAB 2016b	Mathworks	RRID: SCR_001622

## Highlights

- Active Rab6 labels mobile presynaptic cargo in axons of central neurons
- ELKS1 is a Golgin-like protein that captures Rab6 cargo in nerve terminals
- ELKS1 is spread throughout the boutons, and its Rab6 binding is required for capture
- Neurons have re-purposed a cargo-capturing mechanism from constitutive secretion
