# Supplementary material for: ELKS1 Captures Rab6-Marked Vesicular Cargo in Presynaptic Nerve Terminals
Source: Cell Rep. Author manuscript; Available in PMC 2020 Jul 14. (PMC7360120; doi:10.1016/j.celrep.2020.107712)
Supplement: 1 [file NIHMS1603411-supplement-1.pdf]

Cell Reports, Volume *31*

Supplemental Information

ELKS1 Captures Rab6-Marked Vesicular Cargo in Presynaptic Nerve Terminals

Hajnalka Nyitrai, Shan Shan H. Wang, and Pascal S. Kaeser

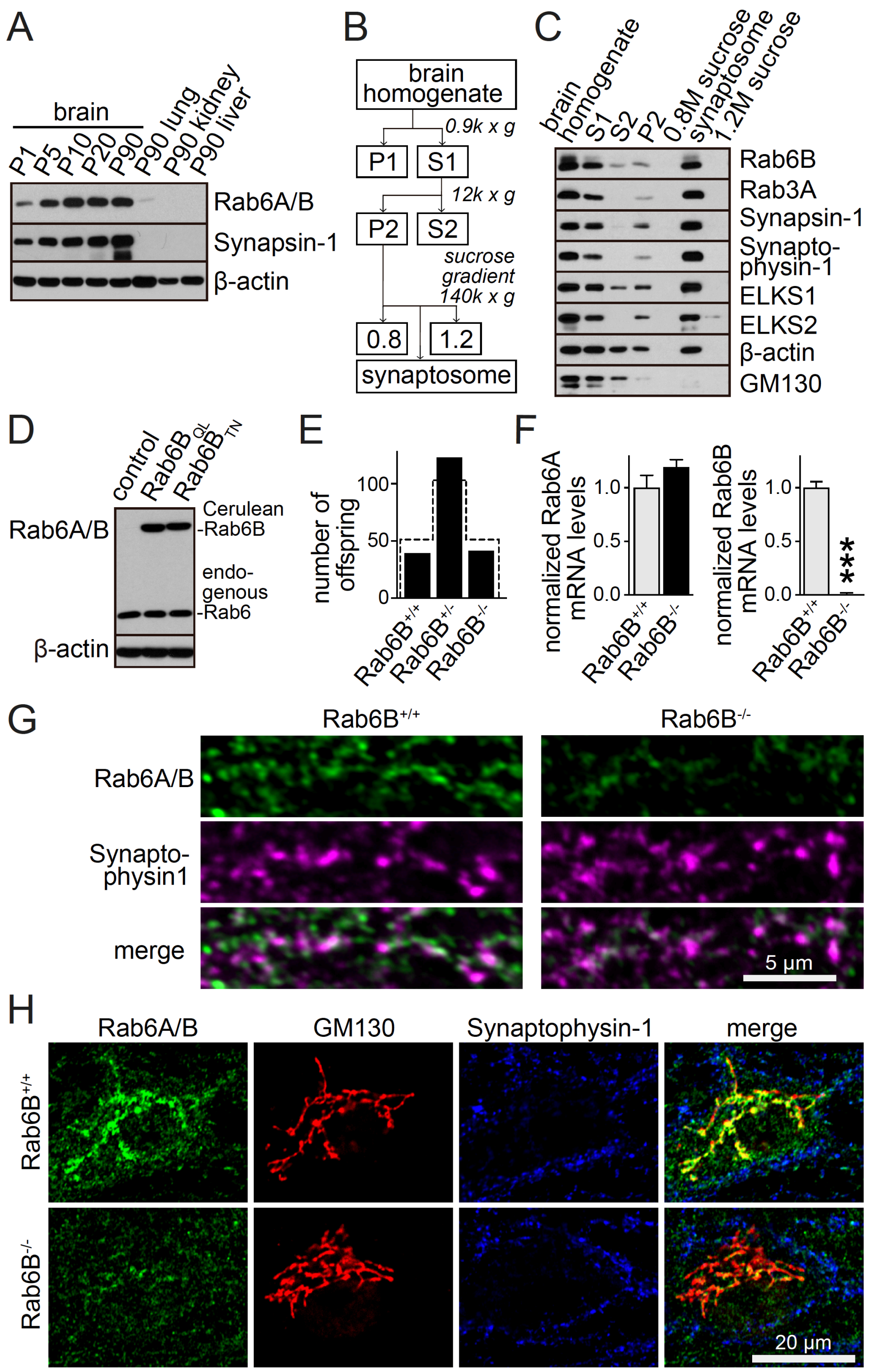

Figure S1. Additional analyses of Rab6 expression and localization, related to Figure 1

(A) Western blotting to assess Rab6 expression in various tissue homogenates. P1-P90 indicates postnatal age of mice at the time of tissue harvest.

(B, C) Schematic representation (B) and western blots (C) of the fractions of a cortical synaptosome preparation of P90 mouse brain tissue, probed with various antibodies.

(D) Western blot to assess expression levels of Cerulean-tagged Rab6B$_{QL}$ or Rab6B$_{TN}$ in

hippocampal cultures transduced with lentiviruses, related to Figs. 1C-1E.

(E) Survival analysis of the offspring of Rab6$^{+/-}$ matings at P14, black bars represent observed offspring numbers, the grey dotted line represents expected offspring numbers. n = 207 mice from 24 litters, related to Figs. 1F-1I.

(F) Real-time quantitative PCR to determine Rab6A and Rab6B mRNA levels in cultured hippocampal neurons of Rab6B$^{-/-}$ and Rab6B$^{+/+}$ littermate mice, related to Figs. 1F-1I, n = 3 independent cultures for each genotype.

(G) Example confocal images of synapse dense areas of cultured hippocampal neurons of Rab6B$^{-/-}$ and Rab6B$^{+/+}$ mice stained with anti-Rab6A/B antibodies and anti-Synaptophysin-1 antibodies to mark synapses, related to Figs. 1F-1I.

(H) Example confocal images of somata of cultured hippocampal neurons of Rab6B$^{-/-}$ and Rab6B$^{+/+}$ mice stained with anti-Rab6A/B antibodies, anti-GM130 antibodies to label the cis-Golgi apparatus, and anti-Synaptophysin-1 antibodies to mark synapses, related to Figs. 1F-1I.

Summary data are means ± SEM, *** $p < 0.001$, analyzed by Student's t-test in F, and by Chi-square test in E (no significant difference was detected compared to the expected Mendelian distribution, dotted gray line).

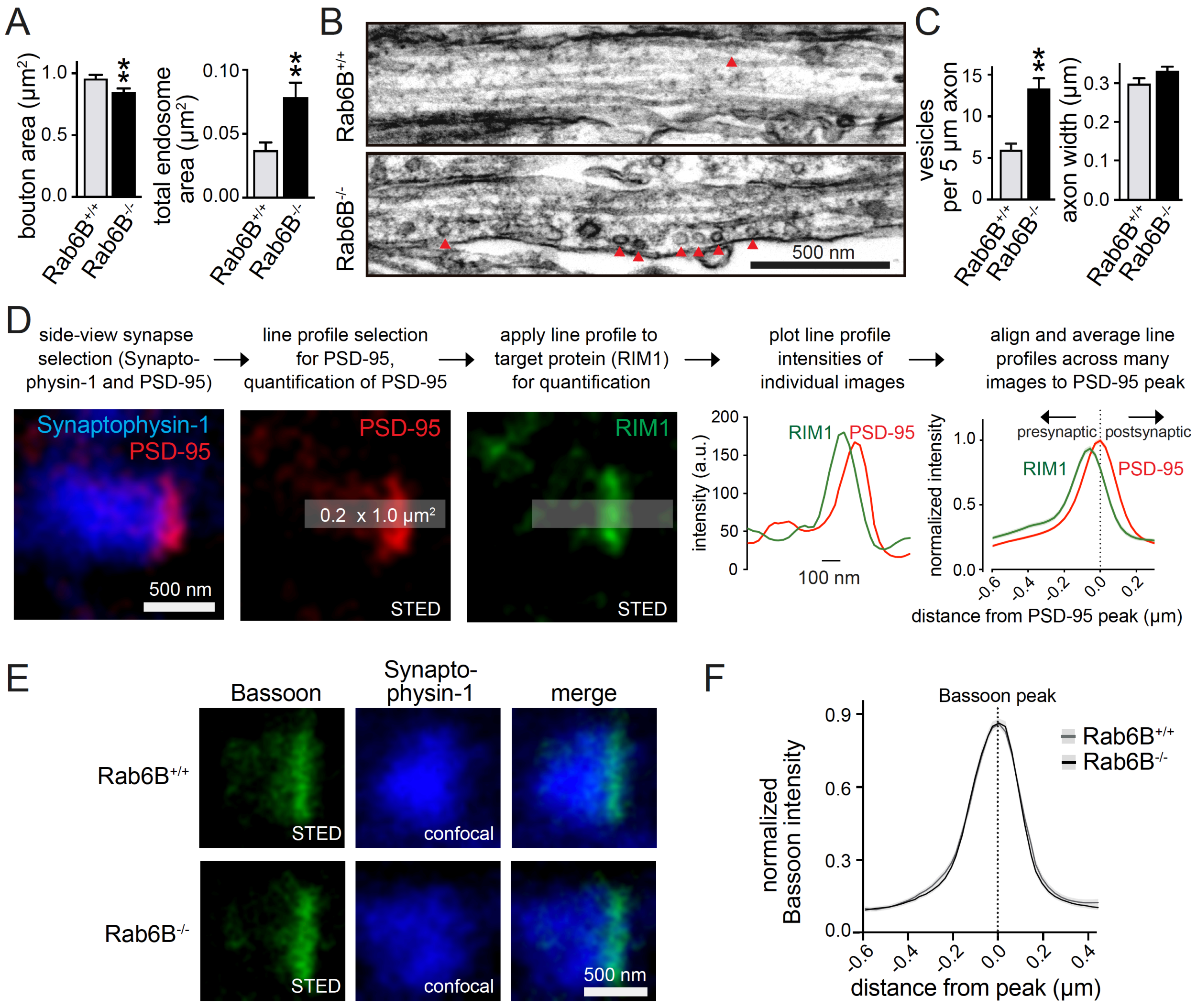

Figure S2. Electron microscopic and STED analyses, related to Figure 2

(A) Analysis of bouton size and endosomal structures in nerve terminals of high-pressure frozen Rab6B$^{+/+}$ and Rab6B$^{-/-}$ neurons, n as in Figs. 2C and 2D.

(B, C) Example electron microscopic images (B) and quantification (C) of axons of Rab6B$^{+/+}$ or Rab6B$^{-/-}$ cultured hippocampal neurons fixed with glutaraldehyde, Rab6B$^{+/+}$, n = 56 axonal segments/1 culture; Rab6B$^{-/-}$, n = 54/1.

(D) Illustration of work flow for STED side-view synapse selection and analysis. The figure repeats the RIM1 and PSD-95 data from Rab6B$^{+/+}$ synapses (including example and summary data) from Figs. 2G and 2H.

(E, F) Example STED images (E) and quantification (F) of Rab6B$^{+/+}$ and Rab6B$^{-/-}$ side-view synapses of hippocampal neurons. Bassoon signals were acquired by STED microscopy, and Synaptophysin-1 signals were acquired by confocal microscopy. F shows normalized intensity profiles of Bassoon signals quantified as described in D, except in the absence of PSD-95 staining, the zero μm position was set to the Bassoon peak. Rab6B$^{+/+}$, n = 47 synapses/3 independent cultures; Rab6B$^{-/-}$, n = 48/3.

Summary data are means ± SEM, ** $p < 0.01$, analyzed by Student's t-test (A and C) or two-way ANOVA (for Bassoon in F: n.s.).

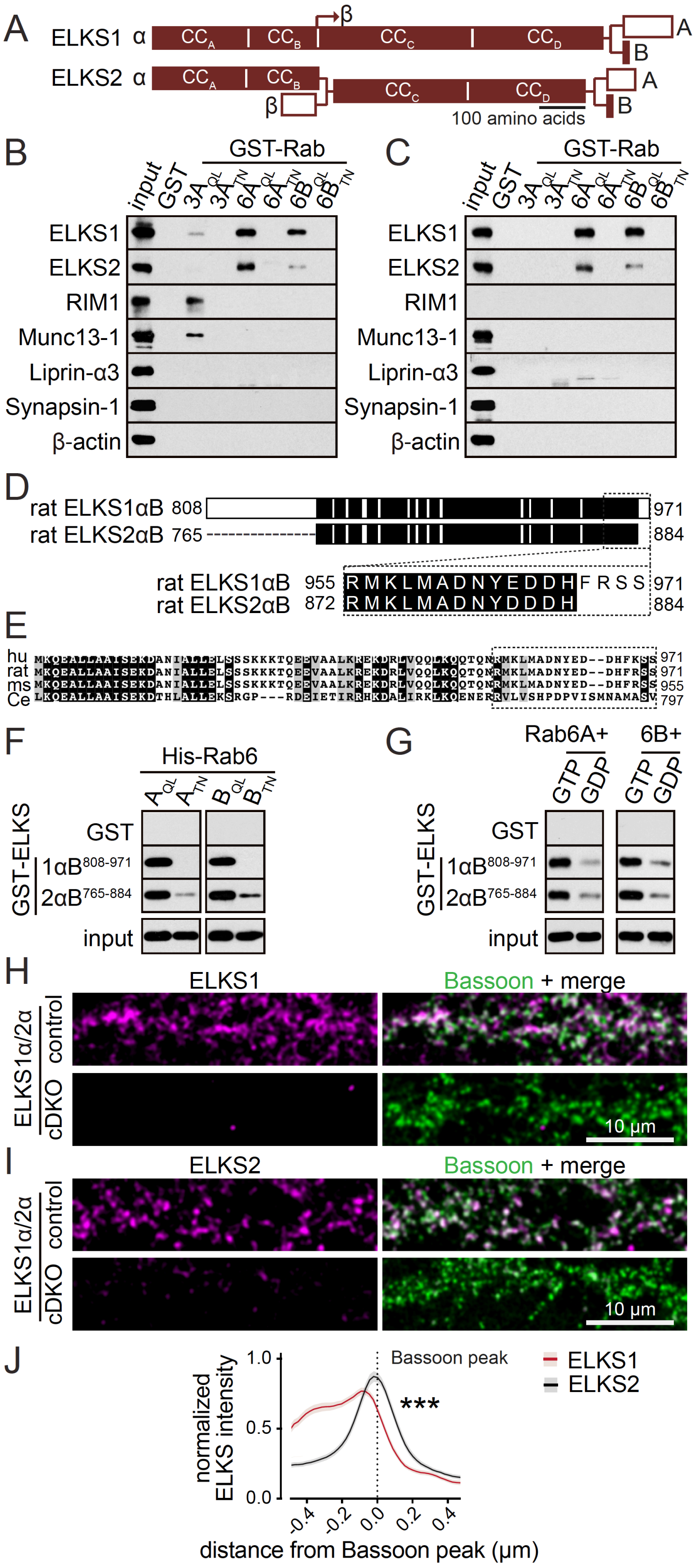

Figure S3. ELKS-Rab6 interaction and ELKS localization, related to Figure 3

(A) Overview of rat ELKS protein isoforms expressed from two genes, *Erc1* and *Erc2*, adapted from (Liu et al., 2014).
(B, C) Example western blots from GST-affinity purifications from brain lysates of adult RIM1α/β$^{+/+}$ (B) and RIM1α/β$^{-/-}$ (C) constitutive knockout mice. GST-tagged Rab proteins were used as baits and interacting proteins were detected by western blotting. The ELKS-Rab6 interaction does not require the presence of RIM1. For GST-Rab6A and -Rab3A baits, n = 3 independent repeats; for GST-Rab6B, n = 1.
(D) Alignment of rat ELKS1 (ELKS1αB$^{808\text{-}971}$, residues LRKKDDR…DDHFRSS, NCBI: XP_017447979) and ELKS2 (ELKS2αB$^{765\text{-}884}$, residues QIEELMN…DNYDDDH, NCBI: AAN39292) protein fragments containing the 17 amino acid stretch that is necessary for Rab6-binding (inset in dotted box). Black shaded areas represent amino acid residues that are 100% conserved, white areas represent lack of residue conservation, and the dotted line indicates absent residues.
(E) Sequence alignment of the C-terminal region of human, rat and mouse ELKS1αB and the corresponding area of C.elegans ELKS. The following sequences were used: human ELKS1αB (MKQEALL…DDHFKSS, Uniprot: Q8IUD2-2), rat ELKS1αB (MKQEALL…DDHFRSS, NCBI: XP_017447979), mouse ELKS1αB (MKQEALL…DDHFRSS, Uniprot: Q99MI1-2), C.elegans ELKS (LKQEALL…MNAMASV, NCBI: NP_500329.1). Black shaded regions indicate 100% sequence homologies. The dotted box marks the 17 amino acid stretch that is required for Rab6-binding of rat ELKS1. Hu = human, ms = mouse, Ce = C.elegans.
(F, G) Western blots of GST-affinity purifications using GST-ELKS proteins to pull down recombinant, His-tagged Rab6. His-Rab6 proteins, which contained a T7 tag at the N-terminus between the His-tag and the Rab6 sequences, were detected by western blotting with anti-T7 antibodies, and input lanes show 5% of total input. In F, active (QL) and inactive (TN) point mutants of Rab6 were used, while in G, Rab6 and either a non-hydrolyzable form of GTP (GMP-PNP) or GDP were used.
(H, I) Example confocal images of ELKS1 (H) and ELKS2 (I) localization at synapses marked by Bassoon in ELKS1α/2α control and ELKS1α/2α cDKO cultured hippocampal neurons, related to Figs. 3E-3K.
(J) Direct comparison of the subsynaptic localization of ELKS1 and ELKS2 side-view control synapses of Figs. 3G and 3J, n as in 3G and 3J.
Summary data are means ± SEM, *** $p < 0.001$, analyzed by two-way ANOVA (J, protein ***, distance ***, interaction ***) followed by Holm-Sidak's post-test at 0 µm, 100 µm, 200 µm, 300 µm, and 400 µm from Bassoon peak, $p < 0.001$ at all points, except at 100 µm, where p is n.s.

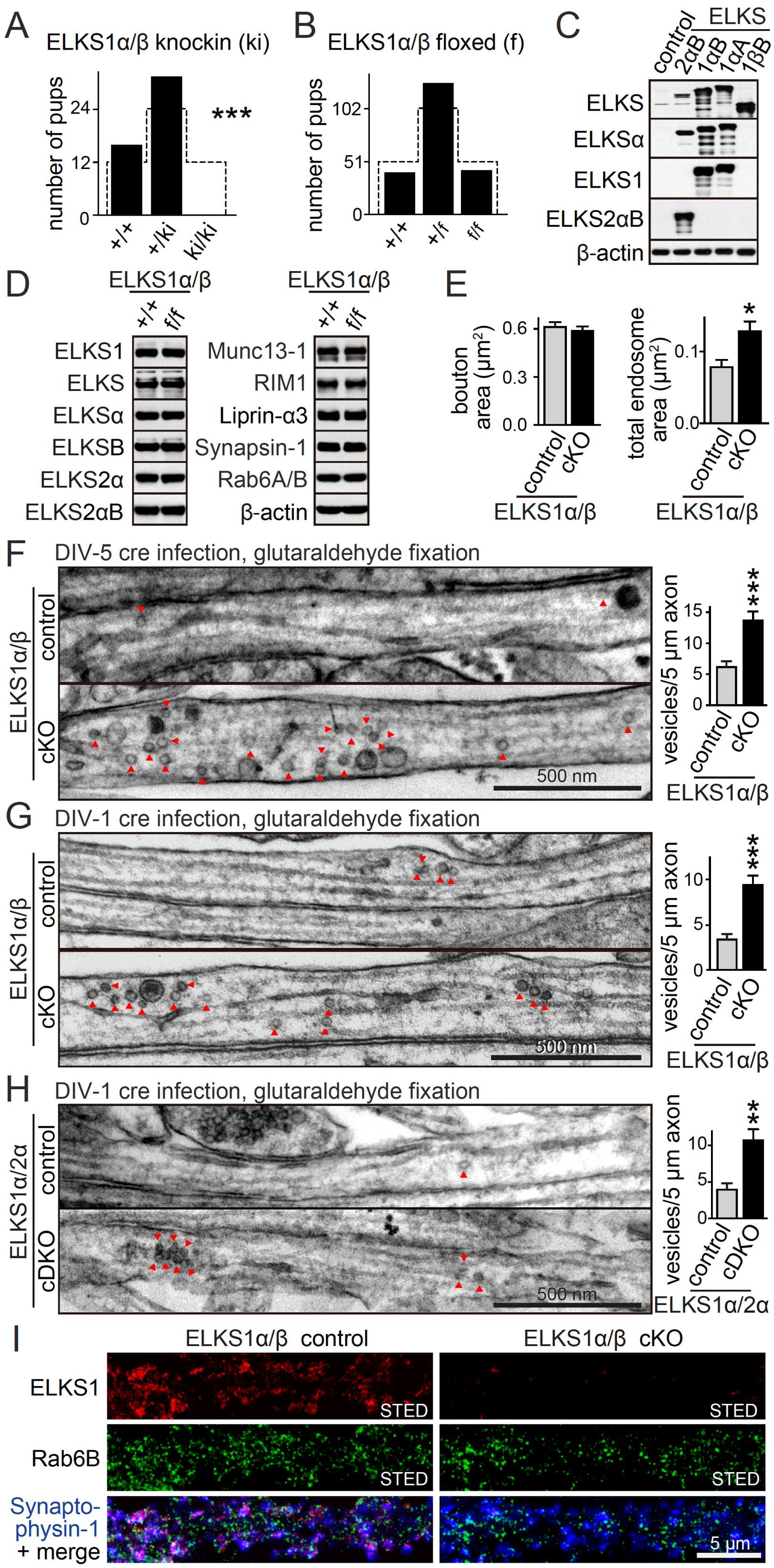

Figure S4. Additional analyses of conditional ELKS1 knockout mice, related to Figure 4

(A, B) Survival analysis of the offspring of matings of the original ELKS1α/β$^{+/ki}$ (A) or ELKS1α/β$^{+/f}$ (B) mice at P14, black bars represent observed offspring numbers. ELKS1α/β$^{ki/ki}$ mice did not survive, while ELKS1α/β$^{f/f}$ mice survived, expected survival is indicated by the grey dotted line, n = 48 mice/8 litters in A, $p < 0.001$, n = 206/27 in B, p: n.s.

(C) Example western blots using the newly generated ELKS antiserum (HM1083) that recognizes all isoforms of ELKS, including the shorter beta (β) isoforms. HEK293T cells were transfected with the various isoforms and cell lysates were subjected to western blotting with HM1083 (top) and other ELKS antibodies used in this study.

(D) Example western blots of brain homogenates of a P30 ELKS1α/β$^{+/+}$ and ELKS1α/β$^{f/f}$ littermate pair using various ELKS and other antibodies. The ELKS1α/β$^{f/f}$ mice express normal levels of ELKS1.

(E) Quantification of bouton size and presynaptic endosomal structures of ELKS1α/β control and ELKS1α/β cKO hippocampal synapses of the experiment shown in Figs. 4D and 4E, n as in Figs. 4D and 4E.

(F, G) Electron microscopic analysis of axons of ELKS1α/β control and ELKS1α/β cKO hippocampal neurons fixed by glutaraldehyde. Neurons were transduced with cre-expressing lentiviruses either at DIV5 (F) or at DIV1 (G). F: ELKS1α/β control, n = 59 axonal segments/1 culture; ELKS1α/β cKO, n = 64/1; G: ELKS1α/β control, n = 51/1; ELKS1α/β cKO, n = 57/1.

(H) Electron microscopic analysis of axons of ELKS1α/2α control and ELKS1α/2α cDKO hippocampal neurons fixed by glutaraldehyde. Neurons were transduced with cre-expressing lentiviruses at DIV1. ELKS1α/2α control, n = 53/1; ELKS1α/2α cDKO, n = 52/1.

(I) Example STED overview images of Rab6B localization at synapses marked by Synaptophysin-1 in ELKS1α/β control and ELKS1α/β cKO cultured hippocampal neurons, related to Figs. 4H-4I.

Summary data are means ± SEM, * $p < 0.05$, ** $p < 0.01$, *** $p < 0.001$, analyzed by Student's t-test in E-H, and by Chi-square test in A and B compared to the expected Mendelian distribution (dotted gray line).

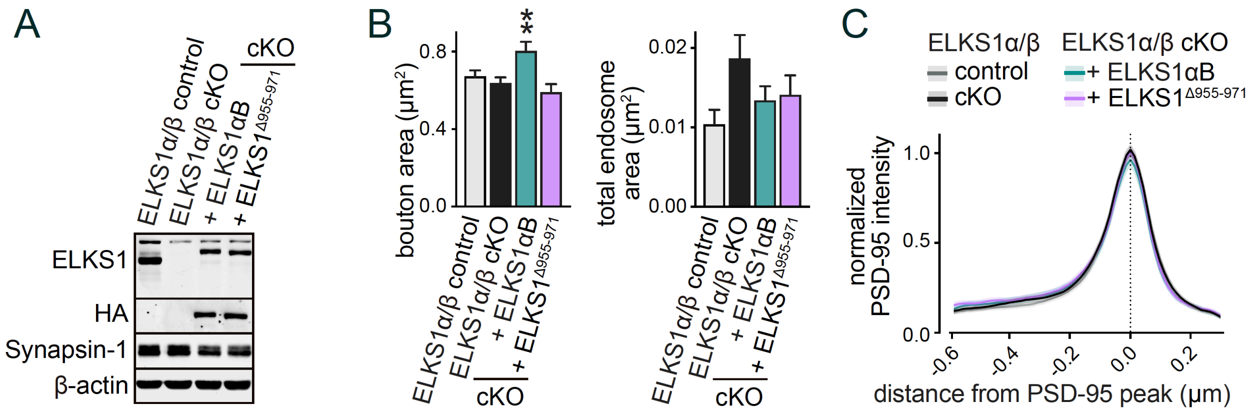

Figure S5. Expression, electron microscopic and PSD-95 localization data for ELKS rescue experiments, related to Figure 5

(A) Example western blots to assess levels of expression of rescue constructs in cultured ELKS1α/β control neurons, ELKS1α/β cKO neurons, and ELKS1α/β cKO neurons transduced with lentiviruses expressing HA-ELKS1αB or HA-ELKS1$^{\Delta 955\text{-}971}$.

(B) Quantification of bouton area and total endosome area in boutons of high-pressure frozen samples presented in Figs. 5A and 5B, n as in Figs. 5A and 5B. The increased endosome area in boutons (Fig. S4E) may not be influenced by ELKS1 binding to Rab6.

(C) Quantification of peak localization and levels of PSD-95 in side-view synapses of the experiment presented in Figs. 5G and 5H, n as in Figs. 5G and 5H.

Summary data are means ± SEM, ** $p < 0.01$, analyzed using one-way ANOVA (B, bouton area ***, endosome area n.s.), followed by Holm-Sidak's post-test (B, comparisons against cKO shown, bouton area), or two-way ANOVA (C, genotype n.s., distance ***, interaction n.s.).

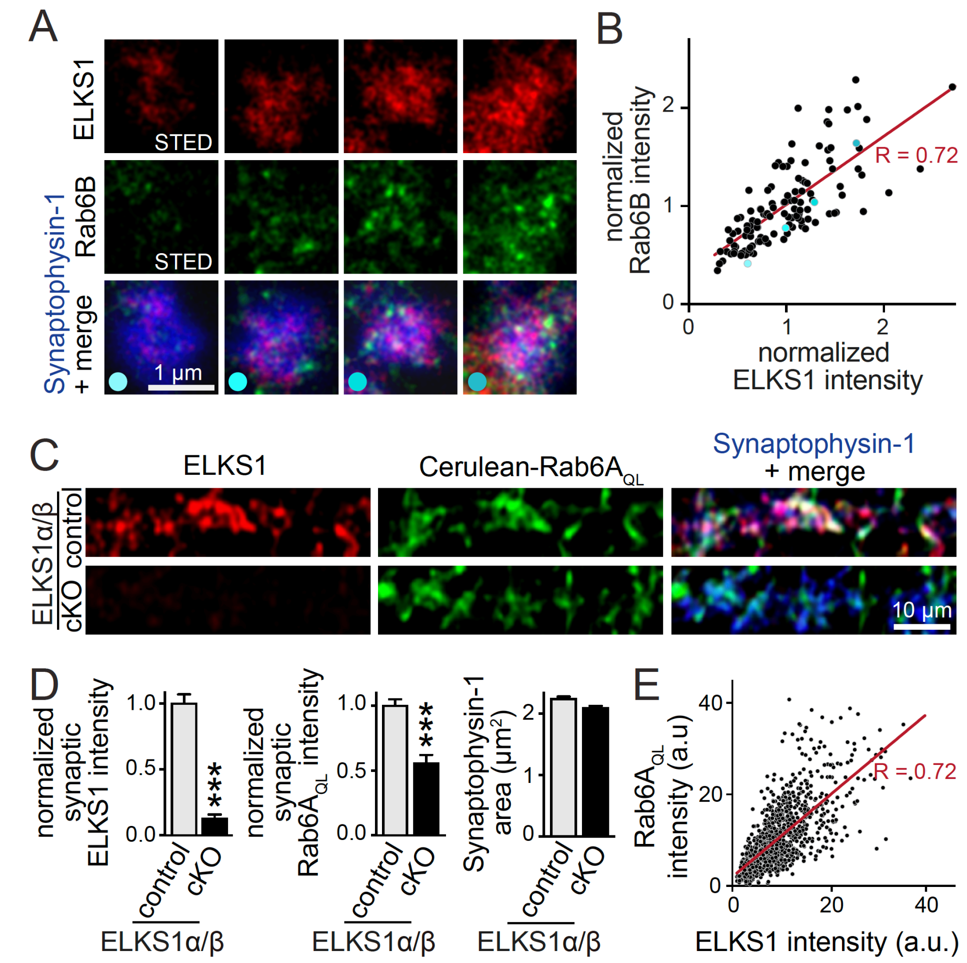

Figure S6. Correlation of synaptic Rab6B and ELKS1 intensities, and synaptic capture of Rab6A$_{QL}$, related to Figure 6

(A, B) Example STED images (A) and quantification of the Pearson's correlation of signal intensities (B) for synaptic Rab6B and ELKS1, intensities were normalized to the average intensity for each signal, n = 162 synapses/3 independent cultures.

(C, D) Example confocal images (C) and quantification (D) of synaptic levels of Cerulean-tagged, transduced Rab6A$_{QL}$ in immunostained ELKS1α/β control and ELKS1α/β cKO neurons. ELKS1α/β control, n = 18 images/3 independent cultures; ELKS1α/β cKO, n = 17/3 (each image containing 37 Synaptophysin-1 objects on average).

(E) Pearson's correlation analysis of Cerulean-Rab6A$_{QL}$ and ELKS1 fluorescent intensities of the ELKS1α/β control condition shown in C and D, n = 1108 synapses/3 independent cultures.

Summary data in D are means ± SEM, *** $p < 0.001$, analyzed by Student's t-test.

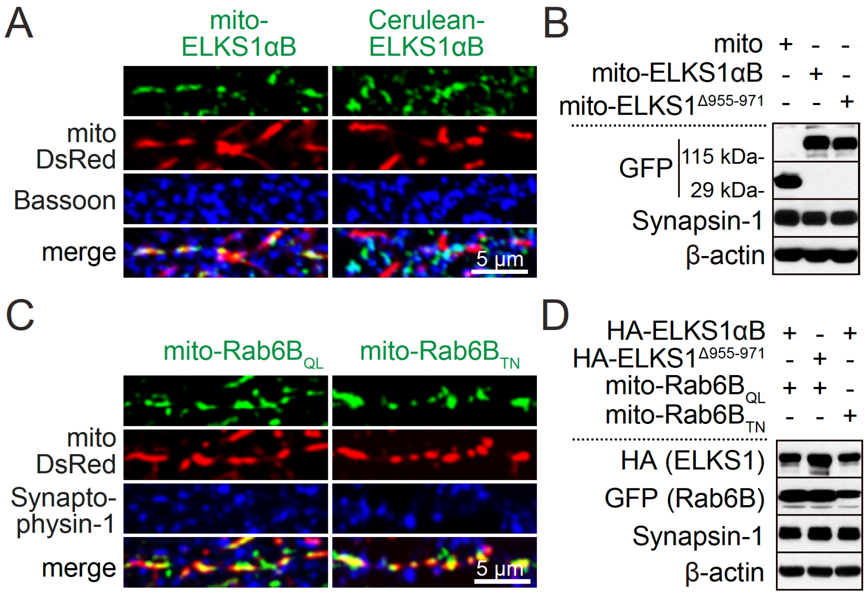

Figure S7. Mitochondrial localization and expression levels of mito-ELKS and mito-Rab6 constructs, related to Figure 7

(A) Example confocal images of cultured hippocampal neurons transduced with lentiviruses expressing either mito-ELKS1αB or ELKS1αB (tagged with Cerulean but without the mitochondrial targeting sequence) and with a second lentivirus expressing mito-DsRed, stained with antibodies against GFP and Bassoon. Mito-ELKS1αB colocalizes with mito-DsRed, while ELKS1αB colocalizes with the synaptic marker Bassoon.

(B) Western blot of cultured neurons transduced with lentiviruses expressing mito-Cerulean, mito-Cerulean-ELKS1αB or mito-Cerulean-ELKS1$^{\Delta 955\text{-}971}$, illustrating overall expression levels of the mito-tagged proteins, related to Figs. 7A-7C.

(C, D) Experiments similar to A and B, but for neurons expressing mito-Rab6 and HA-ELKS constructs, related to Figs. 7D-7F.
